# Ambiphilic Frustrated Lewis Pair Exhibiting High Robustness and Reversible Water Activation: Towards the Metal-Free Hydrogenation of Carbon Dioxide

**DOI:** 10.3390/molecules200711902

**Published:** 2015-06-29

**Authors:** Étienne Rochette, Marc-André Courtemanche, Alexander P. Pulis, Wenhua Bi, Frédéric-Georges Fontaine

**Affiliations:** 1 Department of Chemistry, Centre de Catalyse et Chimie Verte (C3V), Université Laval, 1045 Avenue de la Médecine, Québec, QC G1V 0A6, Canada; E-Mails: etienne.rochette.2@ulaval.ca (É.R.); marc-andre.courtemanche.1@ulaval.ca (M.-A.C.); bi.wenhua.1@ulaval.ca (W.B.); 2 Department of Chemistry, University of Toronto, 80 George Street, Toronto, ON M5S 3H6, Canada; E-Mail: alex.pulis@utoronto.ca

**Keywords:** carbon dioxide, Frustrated Lewis Pairs, hydrogenation, Lewis acid, ambiphilic molecules, catalysis

## Abstract

The synthesis and structural characterization of a phenylene-bridged Frustrated Lewis Pair (FLP) having a 2,2,6,6-tetramethylpiperidine (TMP) as the Lewis base and a 9-borabicyclo[3.3.1]nonane (BBN) as the Lewis acid is reported. This FLP exhibits unique robustness towards the products of carbon dioxide hydrogenation. The compound shows reversible splitting of water, formic acid and methanol while no reaction is observed in the presence of excess formaldehyde. The molecule is incredibly robust, showing little sign of degradation after heating at 80 °C in benzene with 10 equiv. of formic acid for 24 h. The robustness of the system could be exploited in the design of metal-free catalysts for the hydrogenation of carbon dioxide.

## 1. Introduction

Since the seminal discovery by Stephan and coworkers that sterically hindered Lewis pairs, dubbed Frustrated Lewis Pairs (FLPs), could activate molecular hydrogen [1] and act as catalysts for the hydrogenation of various substrates [2,3,4,5,6,7,8,9], there has been an increasing interest in the use of FLPs as metal-free systems for the capture or activation of gases of environmental concern, such as N_2_O [10,11], SO_2_ [12], and CO_2_ [13,14,15,16] as well as in the trapping of reactive intermediates [17,18,19]. Most of this chemistry has been extensively reviewed [20,21,22,23].

The reduction of CO_2_ has attracted much attention in the past few years, especially for the generation of methanol, which is at the core of the methanol economy [24]. Lewis basic organocatalysts have been shown to be reliable catalysts for the hydroboration [25,26,27,28,29] and the hydrosilylation [30,31] of carbon dioxide to generate methanol derivatives. On the other hand, FLPs incorporating strong Lewis acids tend to reduce carbon dioxide only in a stoichiometric fashion [32,33,34] but with adequate tuning can act as efficient catalysts [35,36,37,38,39,40]. The most prominent examples are complexes of general structure 1-B(OR)_2_-2-PR′_2_-C_6_H_4_ (B(OR)_2_ = Bcat, Bpin, B(OMe)_2_; R′ = Ph, *i*Pr, which can catalyze the hydroboration of CO_2_ to generate methoxyboranes with TOF exceeding 900 h^−1^ at 70 °C [33,34,35,36,37]. Although these contributions are of significant academic interest, the hydroboration and the hydrosilylation of carbon dioxide to methoxyboranes and methoxysilanes, respectively, are costly processes without industrial viability, especially when compared to the hydrogenation of carbon dioxide to methanol. Only a handful of transition metal catalyzed homogeneous systems can achieve such transformation, usually with limited activity [41]. Ashley and O’Hare were the first to achieve the substoichiometric hydrogenation of carbon dioxide to methanol using a 2,2,6,6-tetramethylpiperidine (TMP)/B(C_6_F_5_)_3_ FLP system after heating at 160 °C for six days [42], but we demonstrated that ambiphilic derivatives 1-BR_2_-2-NMe_2_-C_6_H_4_ (R = 2,4,6-Me_3_C_6_H_2_ or 2,4,5-Me_3_C_6_H_2_) were able to operate such transformation under much milder conditions [43].

Aminoboranes, first developed by Piers [44] and further studied by Repo and co-workers [6,9,45,46] are therefore FLPs of choice for the reduction of carbon dioxide. However, many of these species exhibit decomposition, notably by protodeborylation, once H_2_ is activated [6,43]. Also possible is the formation of an iminium ion by abstraction of a hydride in the α position to nitrogen [47], which can occur when electron withdrawing groups, such as perfluoroaryls, are present on boron. In our search for an efficient catalyst for carbon dioxide reduction, we were interested in the design of ambiphilic aminoborane molecules where such degradation pathways would be avoided by containing no hydrogen in α position of the nitrogen or aryl groups on boron. Herein we report the synthesis of 1-(BBN)-2-(TMP)-C_6_H_4_ (BBN = 9-borabicyclo[3.3.1]nonane and TMP = 2,2,6,6-tetramethylpiperidine), which exhibits unique robustness.

## 2. Results and Discussion

### 2.1. Synthesis and Characterization of 1-(BBN)-2-(TMP)-C_6_H_4_

**Scheme 1:**
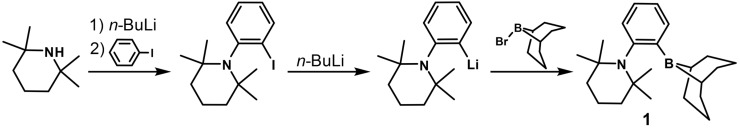
Synthesis of 1-(BBN)-2-(TMP)-C_6_H_4_ (**1**).

The synthesis of species 1-(BBN)-2-(TMP)-C_6_H_4_ (**1**) is illustrated in Scheme 1. It is conveniently prepared by first reacting lithium 2,2,6,6-tetramethylpiperidine with iodobenzene to give 1-I-2-(TMP)-C_6_H_4_. Lithium-halogen exchange between *n*-BuLi and the latter product gave 1-Li-2-(TMP)-C_6_H_4_ [48,49], which was trapped with Br-BBN to give the desired compound **1** in 73% yield.

The ^1^H- and ^13^C{^1^H} NMR spectra of species **1** displays different chemical shifts for the methyl groups pointing away (0.83 ppm) and towards (1.26 ppm) boron, suggesting that rotation around the nitrogen-aryl bond is slow on the NMR timescale. In contrast, the BBN moiety exhibits fast rotation about the boron-aryl bond on the NMR timescale, as evidenced by the observation of only three carbon resonances for the BBN fragment.

**Figure 1:**
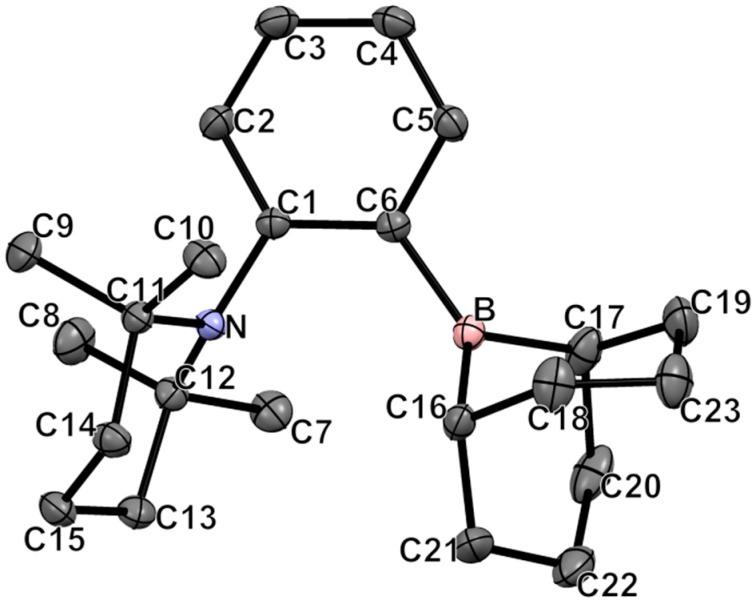
ORTEP diagram of compound **1**. Thermal ellipsoids are drawn at 50% probability. Hydrogen atoms are omitted for clarity. Selected bond lengths (Å) and angles (deg): B-C6 = 1.576(1); N-C1 = 1.442(1); B-N = 3.053(1); C6-B-C16 = 128.25(8); C6-B-C17 = 120.77(8); C16-B-C17 = 110.10(8); C12-N-C11 = 119.27(7); C12-N-C1 = 115.52(7); C11-N-C1 = 115.87(7); N-C1-C6 = 119.67(8); B-C6-C1 = 125.89(8).

Crystals of **1** suitable for single-crystal X-ray diffraction analysis have been grown from hexanes at −35 °C. The structure and the selected bond lengths and bond angles are shown in Figure 1. The sum of internal bond angles around the boron atom (359.2°) is indicative of a trigonal planar conformation. The long B-N distance of 3.053(1) Å indicates no interaction between the Lewis acid and the Lewis base functionalities. Moreover, the X-ray crystal structure of **1** shows that both six-membered cycles of the BBN moiety adopt a chair conformation. The TMP cycle also adopts a chair conformation with two methyl groups pointing toward the boron moiety.

### 2.2. Reactivity of 1-(BBN)-2-(TMP)-C_6_H_4_ with Small Molecules

**Scheme 2:**
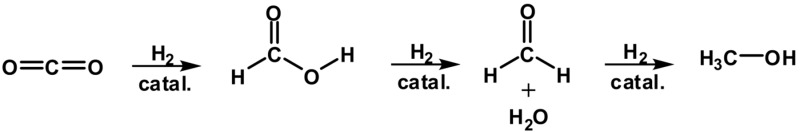
Stepwise hydrogenation of carbon dioxide.

The hydrogenation of carbon dioxide to methanol is a six-electron reduction process that will generate formic acid and formaldehyde as intermediates in addition of generating one equivalent of water, with the end product being methanol, as shown is Scheme 2. A good reduction catalyst therefore needs to show stability towards all of these intermediates and products.

When species **1** is exposed to water, a novel species is observed indicative of the splitting of water (**2**). The ^11^B-NMR signal shifts from 83.4 ppm corresponding to a R_3_B compound for **1** to 0.0 ppm corresponding to a R_3_BOH^−^. The signals of the aliphatic carbons directly linked to the heteroatoms are also consistent with the formation of a zwitterion; the ^13^C{^1^H} NMR signal for C11 and C12, directly linked to the nitrogen, shifts to lower field by 7.6 ppm when those of C16 and C17, directly linked to the boron, shift to higher field by 5.1 ppm. Finally, a resonance is observed in the ^1^H-NMR spectrum at very low field (δ = 17.1) for the splitting of water, which is quite similar to the equivalent resonance, observed by Jäkle, for a pyridylferrocene derivative [50] and suggests hydrogen bonding between O and the proton linked to N. It was found that the water adduct can be reverted back to **1** after a solution of **2** in benzene-*d*_6_ was stored over 4 Å molecular sieves at room temperature for *ca.* 12 h. However, when placed in presence of excess water (10 equiv.) for 12 h, **2** shows signs of hydrolysis.

**Figure 2:**
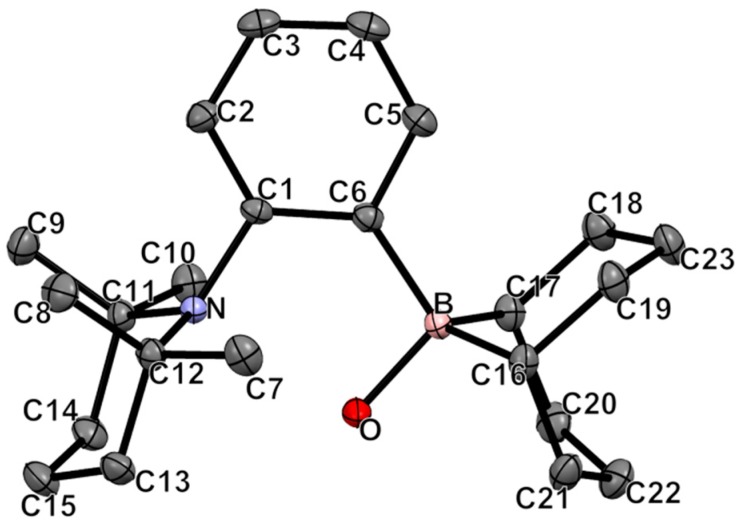
ORTEP diagram of compound **2**. Thermal ellipsoids are drawn at 50% probability. Hydrogen atoms are omitted for clarity. Selected bond lengths (Å) and angles (deg): B-O = 1.539(2); B-C6 = 1.666(2); N-C1 = 1.490(2); B-N = 3.121(2); C6-B-C16 = 114.9(1); C6-B-C17 = 114.9(1); C6-B-O = 105.3(1); C16-B-C17 = 104.2(1); C12-N-C11 = 118.3(1); C12-N-C1 = 114.1(1); C11-N-C1 = 116.1(1); N-C1-C6 = 118.3(1); B-C6-C1 = 126.9(1).

It was possible to obtain crystals of **2** by crystallization from hexanes at −35 °C. The structure is shown in Figure 2. As observed for the crystal structure of **1**, the TMP cycle in **2** adopts a chair conformation with two methyl groups of the TMP framework pointing toward the BBN moiety. The sum of internal bond angles around the nitrogen atom (348.5°) indicates a higher degree of pyramidalization compared to **1**, which is consistent with the coordination of a proton on nitrogen, which was not located in the Fourier map. Moreover, the N-C1 bond is stretched from 1.442(1) in **1** to 1.490(2) Å. The six-membered rings of the borane also adopt a chair conformation. The geometry around boron is tetrahedral, as expected from a borate moiety, with the B-C6 elongated from 1.576(1) on **1** to 1.666(2) Å.

The reaction between formic acid and **1** generated a novel product, **3**, which is reminiscent to the product obtained from the splitting of water. The signals of the aliphatic carbons directly linked to the heteroatoms suggest the formation of a zwitterionic species, as the one observed in **2**. According to the numbering scheme used for the structure of **2**, the signals of C11 and C12, which are directly linked to the nitrogen show a ^13^C-NMR shift to lower field by 14.6 ppm while those of C16 and C17, directly linked to the boron, shift to higher field by 7.4 ppm for **3**. The signals of C22 and C23, which are equivalent on the ^13^C-NMR spectra in **1**, exhibit inequivalence in **3** as observed in the case of **2**. The ^11^B-NMR resonance at δ = 2.1 is also consistent with the formation of a zwitterionic species. The reversibility of the formic acid adduct was also studied and **3** partially (*ca.* 50%) reverted back to **1** after standing over K_2_CO_3_ in a solution of benzene-*d*_6_ at room temperature for *ca.* 12 h. Finally, when placed in presence of excess formic acid (10 equiv.), **3** showed very little degradation (less than 5%) even after heating at 80 °C for 24 h.

Methanol also reacts with **1**, but does not form a stable adduct at room temperature as observed by ^1^H-NMR spectroscopy. The spectrum at room temperature of **1** in CDCl_3_ in presence of *ca.* five equivalents of methanol exhibits broadening of the resonances, in particular for the aromatic protons and for the signal at δ = 1.26 attributed to the methyl group pointing towards the BBN moiety, suggesting a rapid exchange process between **1** and a methanol-bound adduct. However, at lower temperature (−20 °C) signals of a new compound, corresponding to a methanol-bound adduct are present on the ^1^H-NMR spectrum along with those of **1**. At −40 °C, **1** is almost totally converted into a methanol-bound adduct (see SI for details). The steric repulsion between the methyl group of the methanol and the bulky surrounding of the FLP cavity is thought to disfavour the coordination of the bulkier alcohol when compared to water. The reactivity with formaldehyde was studied by placing a benzene-*d*_6_ solution of **1** at 80 °C with excess paraformaldehyde which is known to convert into formaldehyde upon heating. After 12 h, a singlet at 8.67 ppm is observed, indicative of free formaldehyde, but the resonances attributed to **1** remained unchanged. Finally, no reaction was observed when **1** was exposed to up to 80 atm of molecular hydrogen and 1 atm of carbon dioxide at 40 °C.

### 2.3. DFT Study of the Generated Adducts

**Figure 3:**
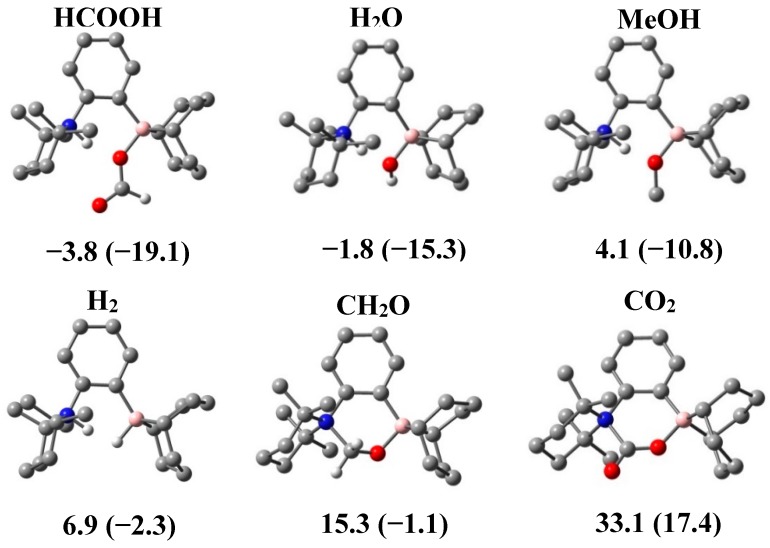
DFT calculations at ωB97XD/6-31++G** level of theory of the adducts of **1** with various small molecules. The ΔG (ΔH) values in kcal∙mol^−1^.

To gain more insight on the stability of various adducts that could be generated between **1** and the molecules of interest, DFT calculations have been carried and the models are exposed in Figure 3 with the ΔG (ΔH) values given in kcal∙mol^−1^. First, it can be observed that the formation of adducts with CO_2_ or formaldehyde are quite endergonic, at 17.4 (33.1) and 15.3 (−1.1) kcal∙mol^−1^. The cleavage of molecular hydrogen is however more favoured, with respective values of 6.9 (−2.3) kcal∙mol^−1^ for the formation of a zwitterionic species. The entropic contribution seems to play a very important role and explains the absence of reactivity between **1** and H_2_, since in terms of enthalpy the reaction should be exothermic at −2.3 kcal∙mol^−1^. Whereas the energy values observed for the methanol adduct justify the presence of a fluxional process (4.1 (−10.8) kcal∙mol^−1^), the free energies observed for the addition of water and formic acid support the formation of novel zwitterionic compounds, with values of −1.8 (−15.3) and −3.8 (−19.1) kcal∙mol^−1^, respectively. Nevertheless, the ΔG values are very close to 0 kcal∙mol^−1^, which justify that these processes can be reversible at higher temperatures.

## 3. Experimental Section

### 3.1. General Information

Unless otherwise specified, all the manipulations were conducted under an inert atmosphere of dinitrogen, using standard Schlenk and glovebox techniques. Reactions were carried either in a sealed J. Young NMR tube, in which case NMR conversions are indicated, or in standard oven dried Schlenk vessels. Benzene-d_6_ was purified by vacuum distillation from Na/K alloy, or by degassing by three subsequent freeze–pump–thaw cycles followed by standing over activated 3 Å molecular sieves. CDCl_3_ was dried by distillation over P_2_O_5_. Anhydrous CO_2_ was purchased from Praxair and used as received. Ultra high purity hydrogen (5.0 grade) was purchased from Praxair and used as received. 1-(2-iodophenyl)-2,2,6,6-tetramethylpiperidine and [2-(2,2,6,6-tetramethylpiperidin-1-yl)phenyl]lithium were synthesized according to literature [48,49].

NMR spectra were recorded on Agilent Technologies NMR spectrometer (Agilent Technologies, Santa Clara, CA, USA) at 500 MHz (^1^H), 125.758 MHz (^13^C), 202.456 MHz (^11^B), and 160.462 MHz, and a Varian Inova NMR AS400 spectrometer (Agilent Technologies, Santa Clara, CA, USA), at 400.0 MHz (^1^H), 100.580 MHz (^13^C), and 128.378 MHz (^11^B). ^1^H-NMR and ^13^C{^1^H}-NMR chemical shifts are referenced to residual protons in deuterated solvent. Multiplicities are reported as singlet (s), broad singlet (s, br) doublet (d), triplet (t), quartet (q), or multiplet (m). Chemical shifts are reported in ppm. Coupling constants are reported in Hz. gHSQC experiments were performed in order to confirm C-H correlations. The numbering scheme follows the one shown in the Figures below. HRMS characterization was possible using an Agilent Technologies 6210 LC Time of Flight Mass Spectrometer using APPI ionization in positive mode. Products in toluene solutions were introduced to the nebulizer by direct injection. FTIR spectra were recorded using a Nicolet Magna 850 Fourier transform infrared spectrometer (Thermo Scientific, Madison, WI, USA) with a liquid nitrogen cooled narrow-band MCT detector using a diamond ATR accessory (Golden Gate, Specac Ltd, London, UK).

### 3.2. Synthesis

*1-(BBN)-2-(TMP)-C_6_H_4_* (**1**). 917 mg of [2-(2,2,6,6-tetramethylpiperidin-1-yl)phenyl]lithium (4.1 mmol) were weighed into a Schlenk flask containing a Teflon coated magnetic stirring bar and dissolved in toluene (*ca.* 15 mL) and cooled down to *ca.* −80 °C using a liquid nitrogen/acetone bath. In a separate Schlenk flask, 4.1 mL (4.1 mmol) of a 1.0 M solution of BBN-Br in dichloromethane was added and the solvent was removed *in vacuo* to be replaced with *ca.* 4 mL of toluene. The solution of BBN-Br was added dropwise to the cold solution of [2-(2,2,6,6-tetramethylpiperidin-1-yl)phenyl]lithium, which was stirred vigorously throughout the addition. The resulting mixture was left to warm to r.t. and left stirring overnight. The decanted solution was filtered to a separated Schlenk flask via cannula. The resulting solution was evaporated to dryness under reduced pressure and further dried at 80 °C under vacuum for 2 h. The residue was then dissolved in hexanes (*ca.* 5 mL). The resulting solution was left at −35 °C for 72 h to allow complete precipitation of the title compound as a white powder (1.01 g, 73% yield). Crystals suitable for X-ray diffraction were grown by slow evaporation of a hexane solution.


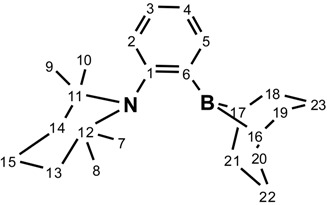


^1^H-NMR 500MHz: δ 7.64 (dd, ^3^*J*_H-H_ = 7.2 Hz, ^4^*J*_H-H_ = 2.0 Hz, 1H, H_2_); 7.39 (dd, ^3^*J*_H-H_ = 7.7 Hz, ^4^*J*_H-__H_ = 1.3 Hz, 1H, H_5_); 7.20 (td, ^3^*J*_H-H_ = 7.3 Hz, ^4^*J*_H-H_ = 1.7 Hz, 1H, H_3_ or H_4_); 7.15 (td, ^3^*J*_H-H_ = 7.3 Hz, ^4^*J*_H-H_ = 1.3 Hz, 1H, H_3_ or H_4_); 2.40 (s, 2H, H_16–17_); 2.13–2.02 (m, 10H, H_18–21_ and H_22,23_); 1.84–1.75 (m, 1H, H_15_); 1.63 (ddd, ^2^*J*_H-H_ = 12.8 Hz, ^3^*J*_H-H_ = 7.8 Hz, ^3^*J*_H-H_ = 2.7 Hz 2H, H_13–14_); 1.56–1.50 (m, 2H, H_13,14_); 1.50–1.43 (m, 1H, H_15_); 1.41 (dt, ^2^*J*_H-H_ = 12.7 Hz, ^3^*J*_H-H_ = 3.4 Hz, 2H, H_22,23_); 1.26 (s, 6H, H_7,10_ or H_8,9_); 0.83 (s, 6H, H_7,10_ or H_8,9_). ^13^C {^1^H} (126 MHz): δ 150.4 (s, 1C, C_1_); 133.0 (s, 1C, C_2_); 131.3 (s, 1C, C_5_); 129.2 (s, 1C, C_3_ or C_4_); 125.0 (s, 1C, C_3_ or C_4_); 54.7 (s, 2C, C_11,12_); 41.4 (s, 2C, C_13,14_); 35.0 (s, 4C, C_18–21_); 34.1 (s, 2C, C_7,10_ or C_8,9_); 32.5 (s, broad 2C, C_16,17_); 25.7 (s, 2C, C_7,10_ or C_8,9_); 23.5 (s, 2C, C_22,23_); 18.6 (s, 1C, C_15_). ^11^B {^1^H} (160 MHz): δ 83.4 (s, 1B). [M + H]^+^, calculated 338.3019; found = 338.2852.

*1-(BBN)-2-(TMP)-C_6_H_4_ H_2_O adduct* (**2**). **2** crystallized out of a solution of **1** exposed to air from hexane and the characterisation was carried out on the few crystals obtained. However, attempts to form **2** in good yield from **1** by adding stoichiometric equivalent of water gave a mixture of **2** and another product that was identified as [2-(2,2,6,6-tetramethylpiperidin-1-yl)phenyl]boronic acid.


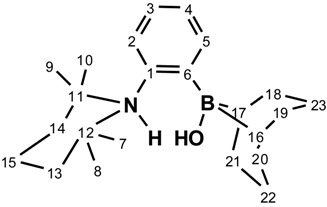


^1^H-NMR 500MHz: δ 17.06 (s, 1H, N-H); 8.49 (d, ^3^*J*_H-H_ = 7.9 Hz, 1H, H_2_); 7.25 (t, ^3^*J*_H-H_ = 7.5 Hz, 1H, H_3_ or H_4_); 6.96 (t, ^3^*J*_H-H_ = 7.5 Hz, 1H, H_3_ or H_4_); 6.80 (d, ^3^*J*_H-H_ = 8.2 Hz, 1H, H_5_); 3.02–2.91 (m, 2H, H_18–__19_ or H_20–21_); 2.61–2.45 (m, 2H, H_22_ or H_23_); 2.42–2.17 (m, 7H, H_18–19_ and H_20–21_ and H_22_ or H_23_); 1.98 (dt, ^2^*J*_H-H_ = 13.4 Hz, ^3^*J*_H-H_ = 6.6 Hz 1H, H_22_ or H_23_); 1.64 (s, 1H, O-H); 1.50 (td, ^2^*J*_H-H_ = 13.6 Hz, ^3^*J*_H-H_ = 3.1 Hz, 2H, H_13–14_); 1.29 (dtt, ^2^*J*_H-H_ = 13.5 Hz, ^3^*J*_H-H_ = 10.5 Hz, ^3^*J*_H-H_ = 3.2 Hz 1H, H_15_); 1.17–1.02 (m, 3H, H_15_ and H_16,17_); 1.06 (dt, ^2^*J*_H-H_ = 14.3 Hz, ^3^*J*_H-H_ = 3.1 Hz, 2H, H_13–14_); 0.95 (s, 6H, H_7,10_ or H_8,9_); 0.93 (s, 6H, H_7,10_ or H_8,9_). ^13^C {^1^H} (126 MHz): δ 140.4 (s, 1C, C_1_); 136.8 (s, 1C, C_2_); 126.1 (s, 1C, C_3_ or C_4_); 123.1 (s, 1C, C_3_ or C_4_); 122.6 (s, 1C, C_5_); 62.3 (s, 2C, C_11,12_); 38.6 (s, 2C, C_13,14_); 33.4 (s, 2C, C_18,19_ or C_20,21_); 32.1 (s, 2C, C_18,19_ or C_20,21_); 29.7 (s, 2C, C_7,10_ or C_8,9_); 27.4 (s, broad 2C, C_16,17_); 24.8 (s, 1C, C_22_ or C_23_); 24.7 (s, 1C, C_22_ or C_23_); 23.7 (s, 2C, C_7,10_ or C_8,9_); 16.7 (s, 1C, C_15_). ^11^B {^1^H} (160 MHz): δ 0.0 (s, 1B).

*1-(BBN)-2-(TMP)-C_6_H_4_ HCOOH adduct* (**3**). 1.00 g of **1** (3.0 mmol) was dissolved in hexanes (*ca.* 15 mL) and 125 μL (1.1 equiv., 3.3 mmol) of formic acid were added provoking the precipitation of **3**. After filtration, the white solid was dried under vacuum and 435 mg of **3** (38% yield) were obtained.


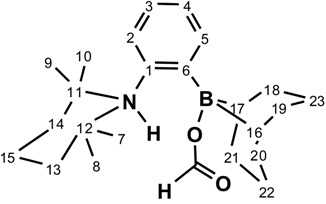


^1^H-NMR 500MHz: δ 8.65 (s, 1H,COOH); 8.01 (dd, ^3^*J*_H-H_ = 7.7 Hz, ^4^*J*_H-H_ = 1.6 Hz, 1H, H_2_); 7.55 (s, broad 1H, N-H); 7.10 (ddd, ^3^*J*_H-H_ = 7.7 Hz, ^3^*J*_H-H_ = 7.2 Hz, ^4^*J*_H-H_ = 1.1 Hz, 1H, H_3_ or H_4_); 6.83 (ddd, ^3^*J*_H-__H_ = 8.2 Hz, ^3^*J*_H-H_ = 7.2 Hz, ^4^*J*_H-H_ = 1.6 Hz, 1H, H_3_ or H_4_); 6.60 (dd, ^3^*J*_H-H_ = 8.2 Hz, ^4^*J*_H-H_ = 1.0 Hz, 1H, H_5_); 2.50–2.40 (m, 2H, H_18–19_ or H_20–21_); 2.28 (m, 1H, H_22_ or H_23_); 2.18–2.11 (m, 1H, H_22_ or H_23_); 2.10–1.95 (m, 6H, H_18–19_ and H_20–21_); 1.91 (td, ^2^*J*_H-H_ = 14.3 Hz, ^3^*J*_H-H_ = 7.4 Hz, 1H, H_22_ or H_23_); 1.75–1.67 (m, 1H, H_22_ or H_23_); 1.40 (s, 2H, H_16–17_); 1.36 (s, 6H, H_7,10_ or H_8,9_); 1.23–1.05 (m, 3H, H_13,14_ and H_15_); 0.91–0.87 (m, 1H, H_15_); 0.83 (dt, ^2^*J*_H-H_ = 15.0 Hz, ^3^*J*_H-H_ = 3.3 Hz 2H, H_13–14_); 0.66 (s, 6H, H_7,10_ or H_8,9_). Some formic acid was also present: 7.84 ppm (s, **H**COOH); 10.61 (s, broad, HCOOH). ^13^C {^1^H} (126 MHz): δ 169.4 (s, 1C, COOH); 142.8 (s, 1C, C_1_); 137.1 (s, 1C, C_2_); 127.9 (s, 1C, C_3_ or C_4_); 124.9 (s, 1C, C_3_ or C_4_); 121.1 (s, 1C, C_5_); 69.3 (s, 2C, C_11.12_); 35.2 (s, 2C, C_13,14_); 32.2 (s, 2C, C_18,19_ or C_20,21_); 31.3 (s, 2C, C_18,19_ or C_20,21_); 30.2 (s, 2C, C_7,10_ or C_8,9_); 27.8 (s, 2C, C_7,10_ or C_8,9_); 25.1 (s, broad, 2C, C_16,17_); 24.5 (s, 1C, C_22_ or C_23_); 23.3 (s, 1C, C_22_ or C_23_); 14.8 (s, 1C, C_15_). Some formic acid was also present at 162.4 ppm.

^11^B {^1^H} (160 MHz): δ 2.1 (s, 1B). [M-HCOO]^+^, calc = 338.3019, found = 338.2612.

## 4. Conclusions

The synthesis and the structural characterization of species 1-(BBN)-2-(TMP)-C_6_H_4_ (**1**) was carried out. The latter molecule exhibits FLP-like reactivity and can split water, formic acid, and methanol at low temperature to generate the respective zwittterionic species, which were fully characterized. There was no reactivity observed with formaldehyde, hydrogen or carbon dioxide, therefore precluding CO_2_ hydrogenation. The DFT calculations involving all reagents and products that should be observed in the hydrogenation of carbon dioxide indicate that with the right tuning of the steric and electronic effects on boron and nitrogen, it would be possible to have a system where reversible formation of adducts is possible, which would make possible the catalytic reduction of carbon dioxide into methanol. We are currently investigating analogues, which should exhibit higher reactivity and enable such transformations.

## Supplementary Materials

The supplementary materials include the NMR and the FT-IR spectra of compounds synthesized, the DFT data and important crystallographic parameters. Crystallographic data have been deposited with CCDC (CCDC No. 1060045 for **1** and CCDC No. 10060046 for **2**). These data can be obtained upon request from the Cambridge Crystallographic Data Centre, 12 Union Road, Cambridge CB2 1EZ, UK, E-Mail: deposit@ccdc.cam.ac.uk, or via the internet at www.ccdc.cam.ac.uk. Supplementary materials can be accessed at: http://www.mdpi.com/1420-3049/20/07/11902/s1.
