# Supplementary material for: Ambiphilic Frustrated Lewis Pair Exhibiting High Robustness and Reversible Water Activation: Towards the Metal-Free Hydrogenation of Carbon Dioxide
Source: Molecules. 2015 Jun 29;20(7):11902–14. doi: 10.3390/molecules200711902 (PMC6331835; doi:10.3390/molecules200711902)
Supplement: Supplementary file 1 [file molecules-20-11902-s001.pdf]

Supporting Information

S1 NMR Spectra

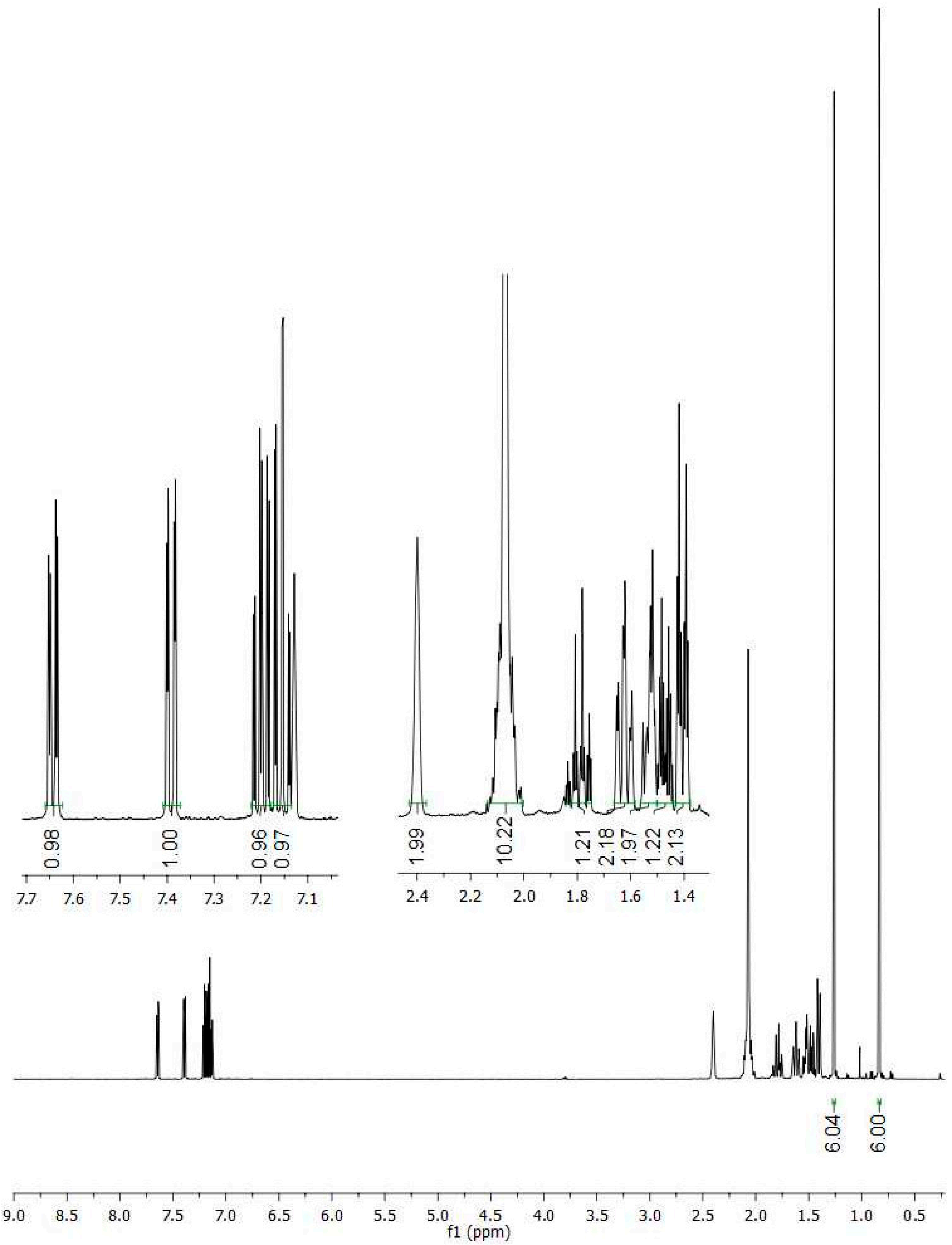

Figure S1. ^{1}H-NMR spectrum (500 MHz, benzene-d_6) of **1**.

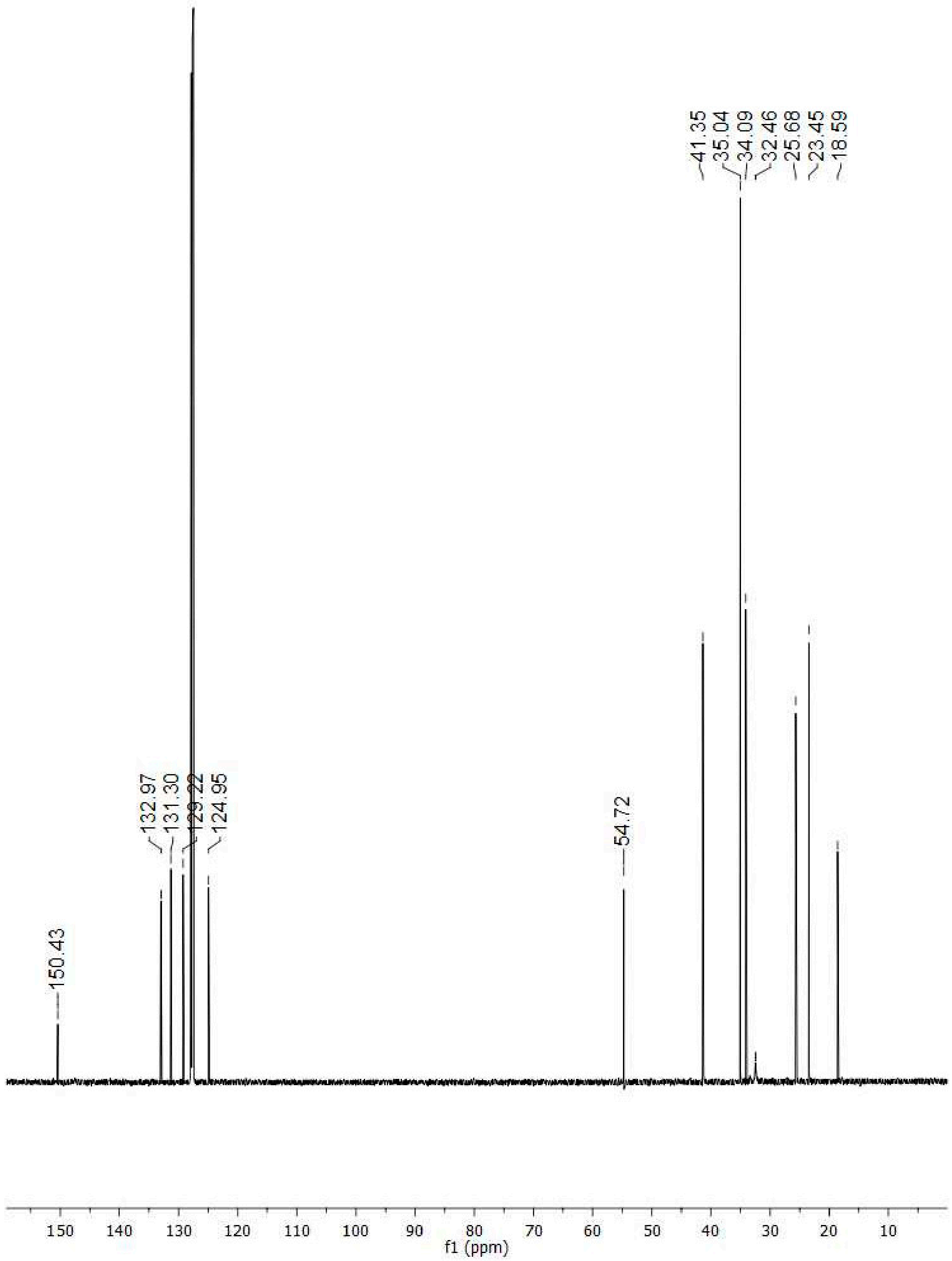

Figure S2. ^{13}C {^{1}H} NMR spectrum (126 MHz, benzene-d_6) of **1**.

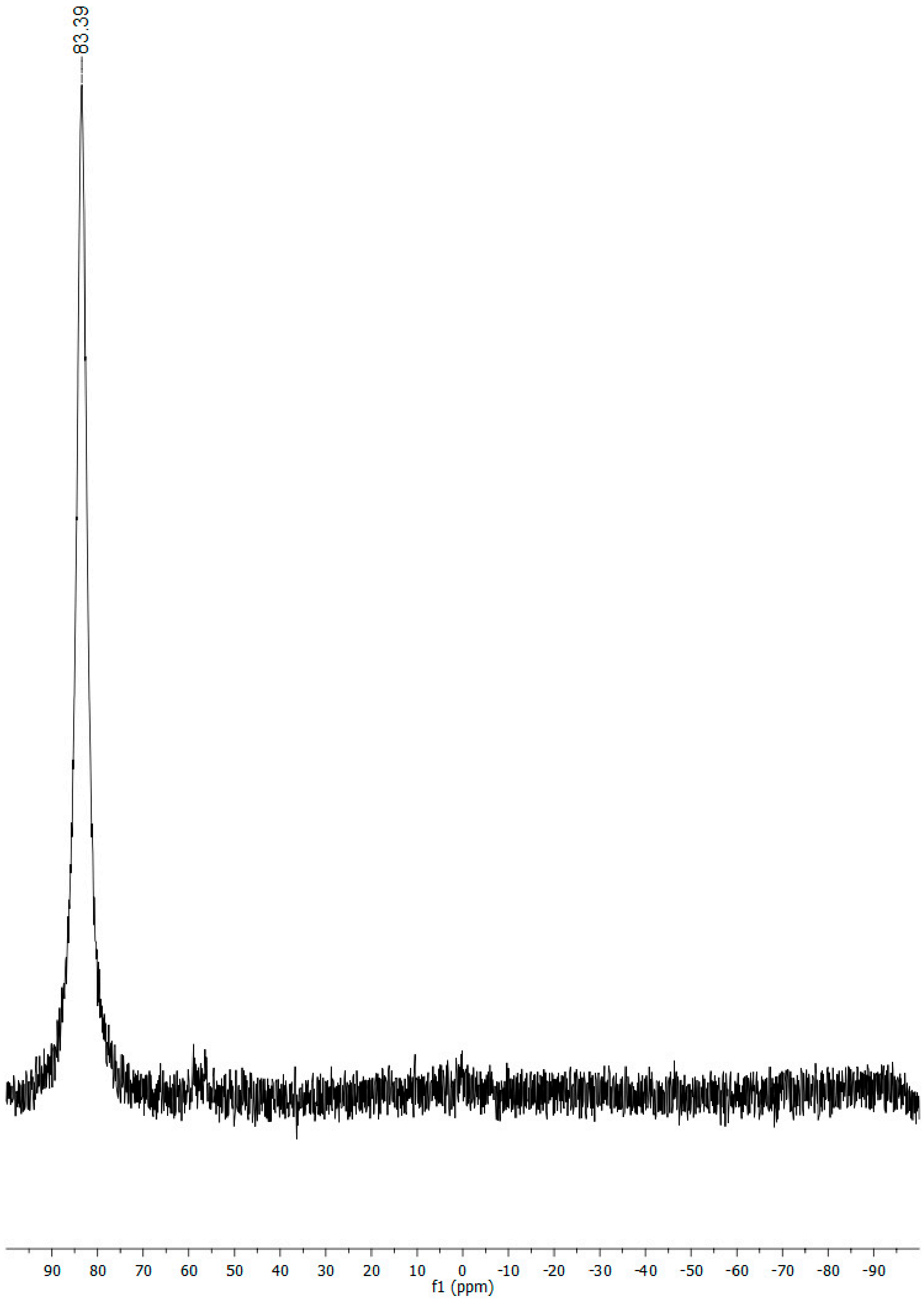

Figure S3. $^{11}B\{^1H\}$ NMR spectrum (160 MHz, benzene-d_6) of **1**.

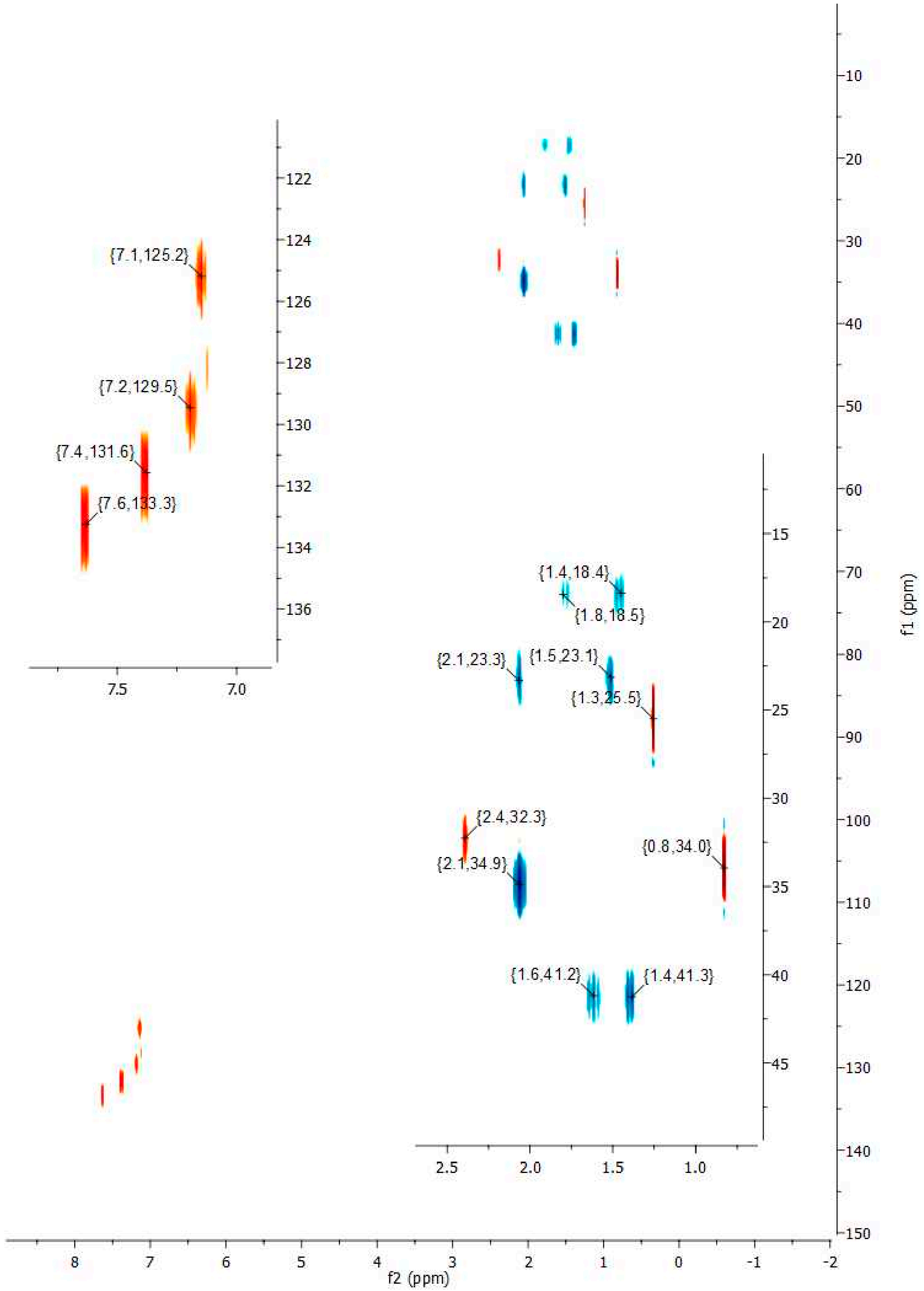

Figure S4. ^{13}C-^{1}H HSQC NMR spectrum (126 MHz–500 MHz, benzene-d_6) of **1**.

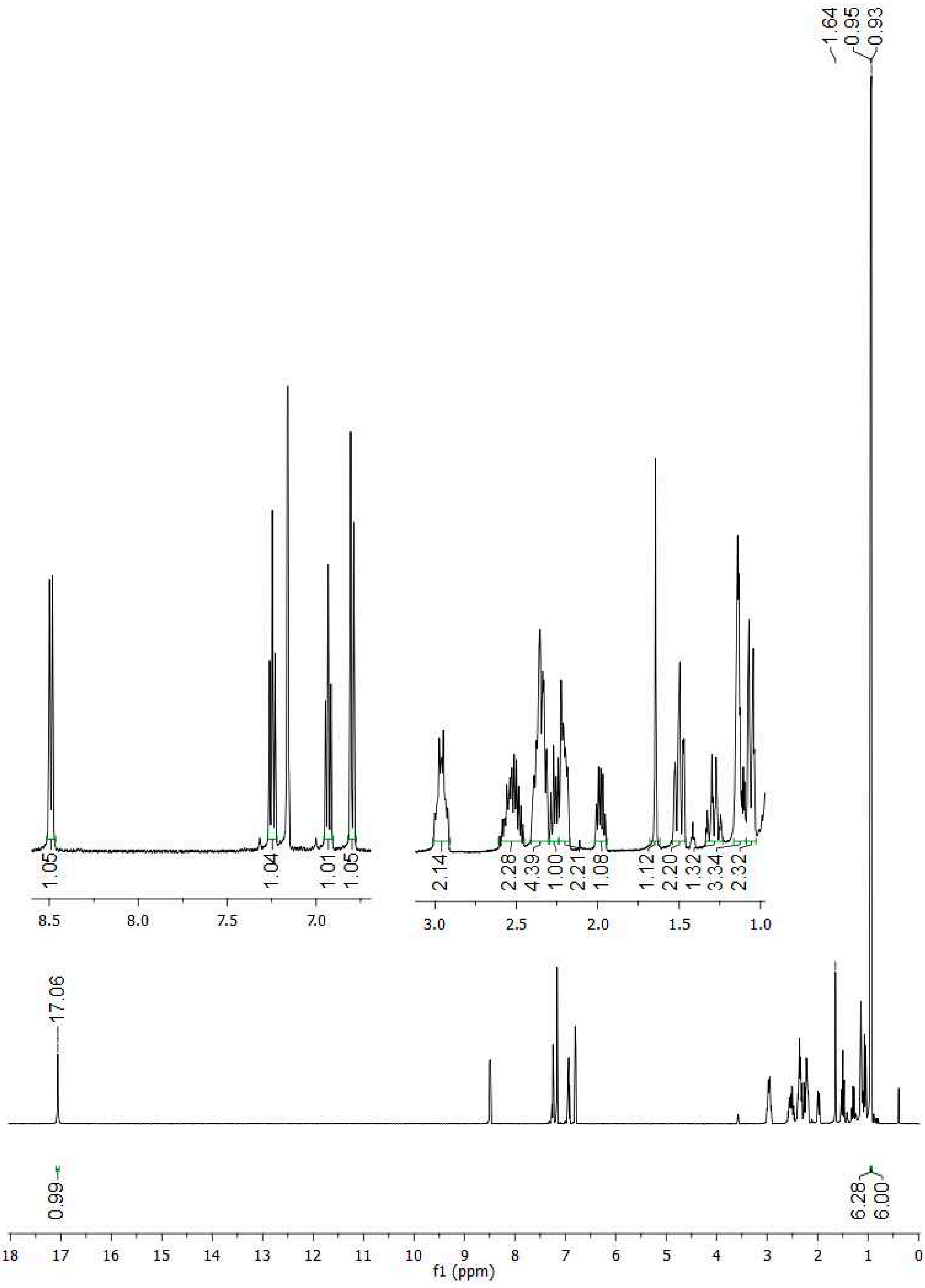

Figure S5. ^{1}H-NMR spectrum (500 MHz, benzene-d_6) of **2**.

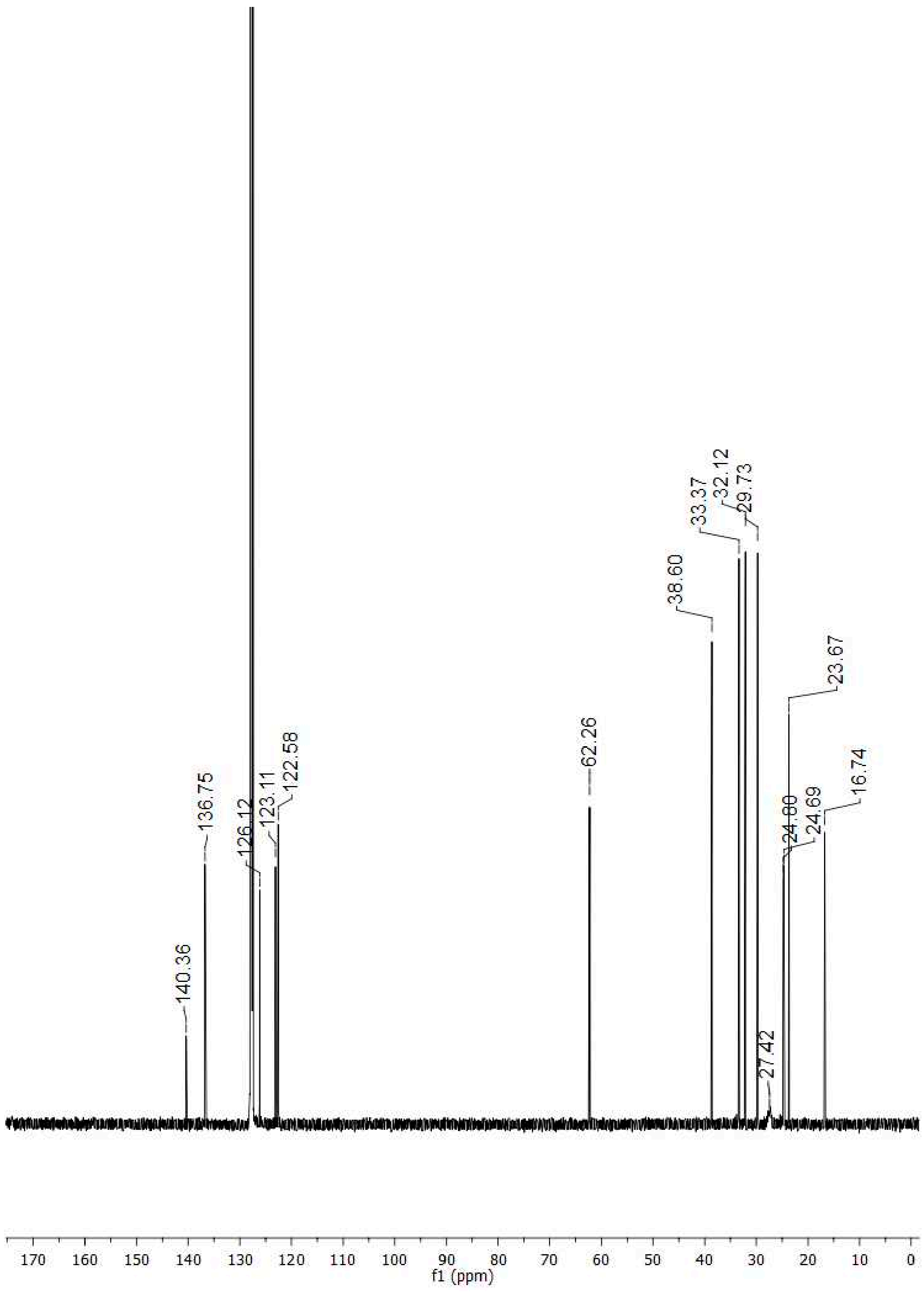

Figure S6. $^{13}C\{^{1}H\}$ NMR spectrum (126 MHz, chloroform-*d*) of **2**.

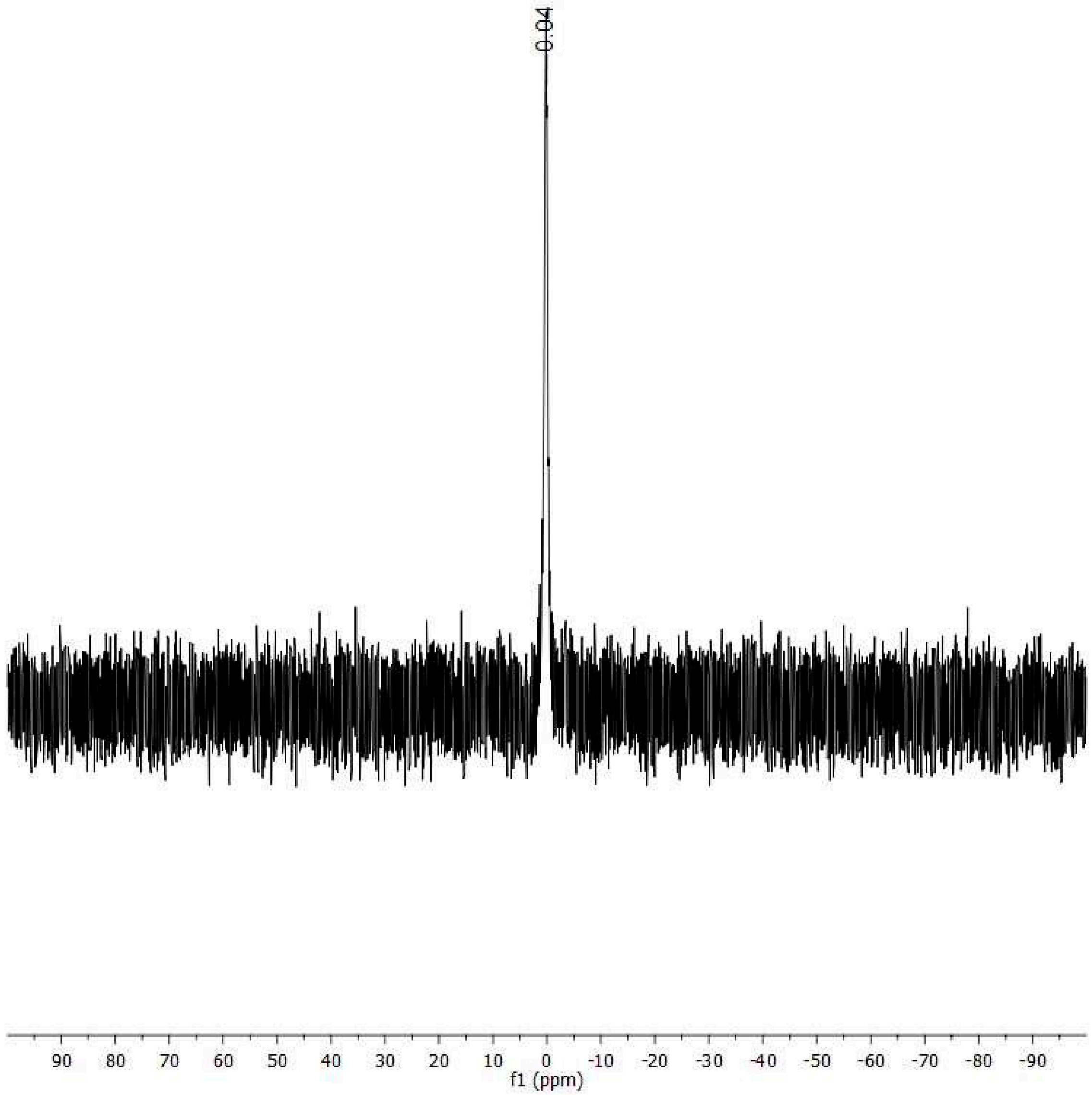

Figure S7. $^{11}B\{^{1}H\}$ NMR spectrum (160 MHz, benzene-d_6) of **2**.

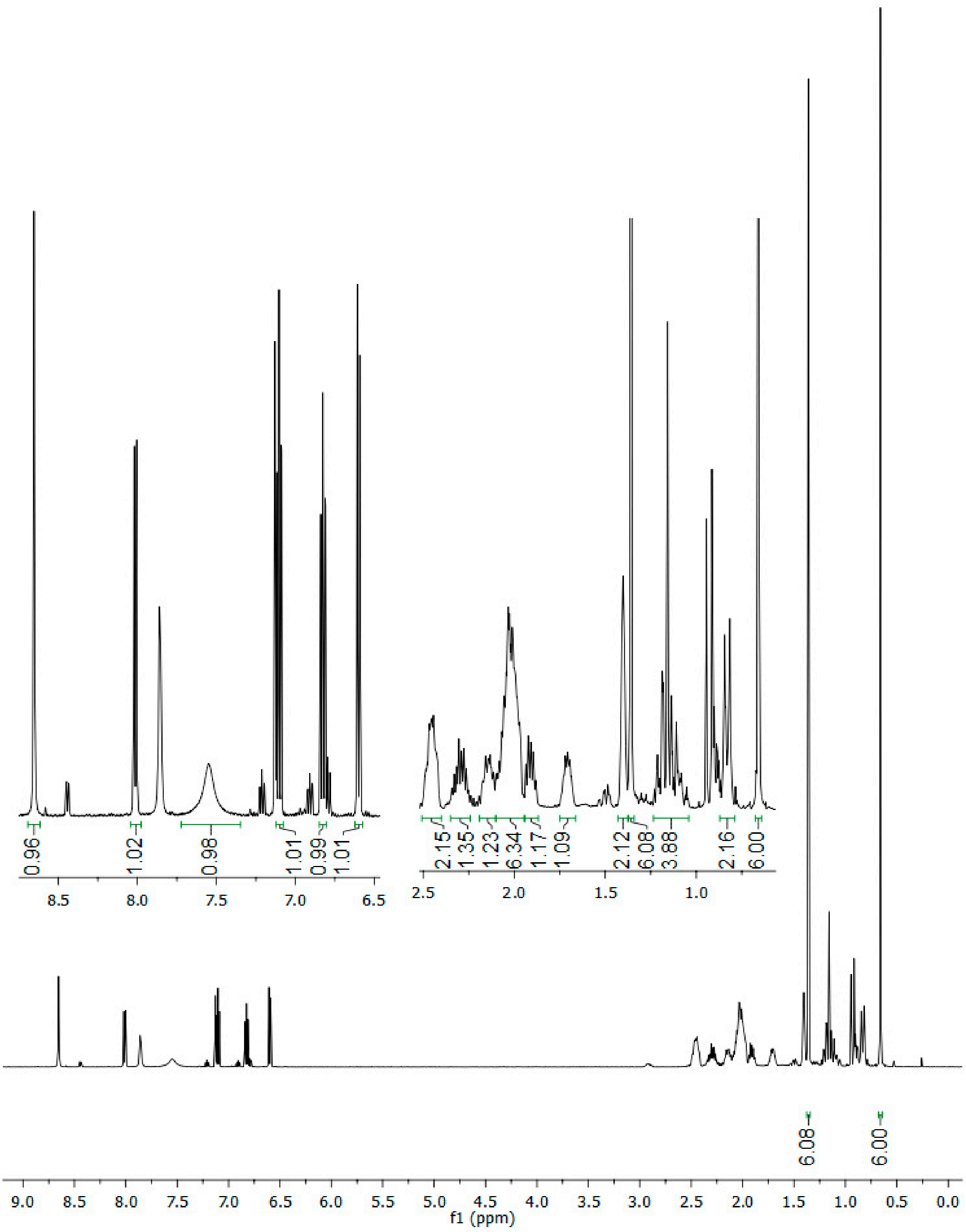

Figure S8. ^{1}H NMR spectrum (500 MHz, benzene-d_6) of **3**.

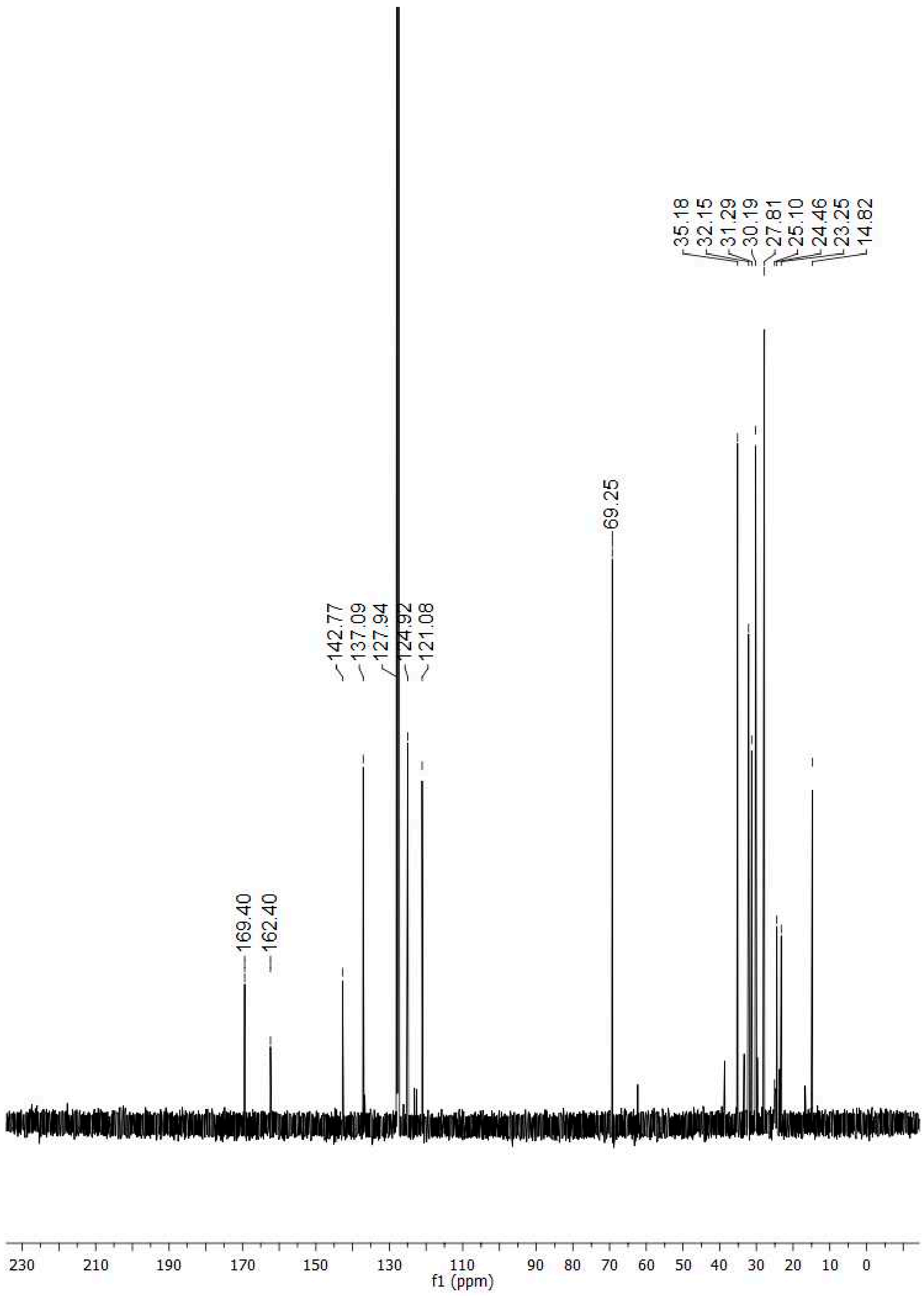

Figure S9. $^{13}C\ \{^{1}H\}$ NMR spectrum (126 MHz, benzene-d_6) of **3**.

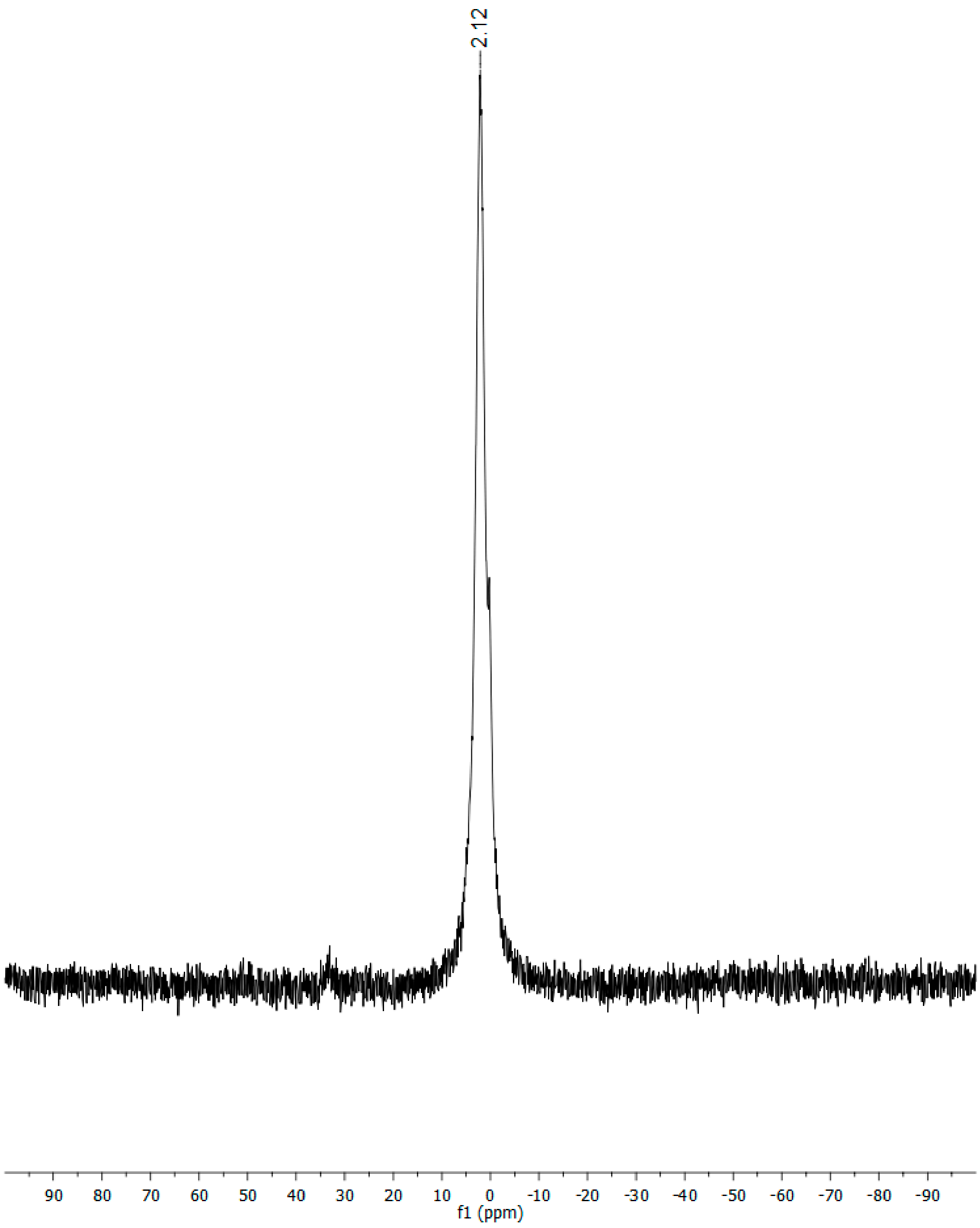

Figure S10. $^{11}B\{^{1}H\}$ NMR spectrum (160 MHz, benzene-*d*6) of **3**.

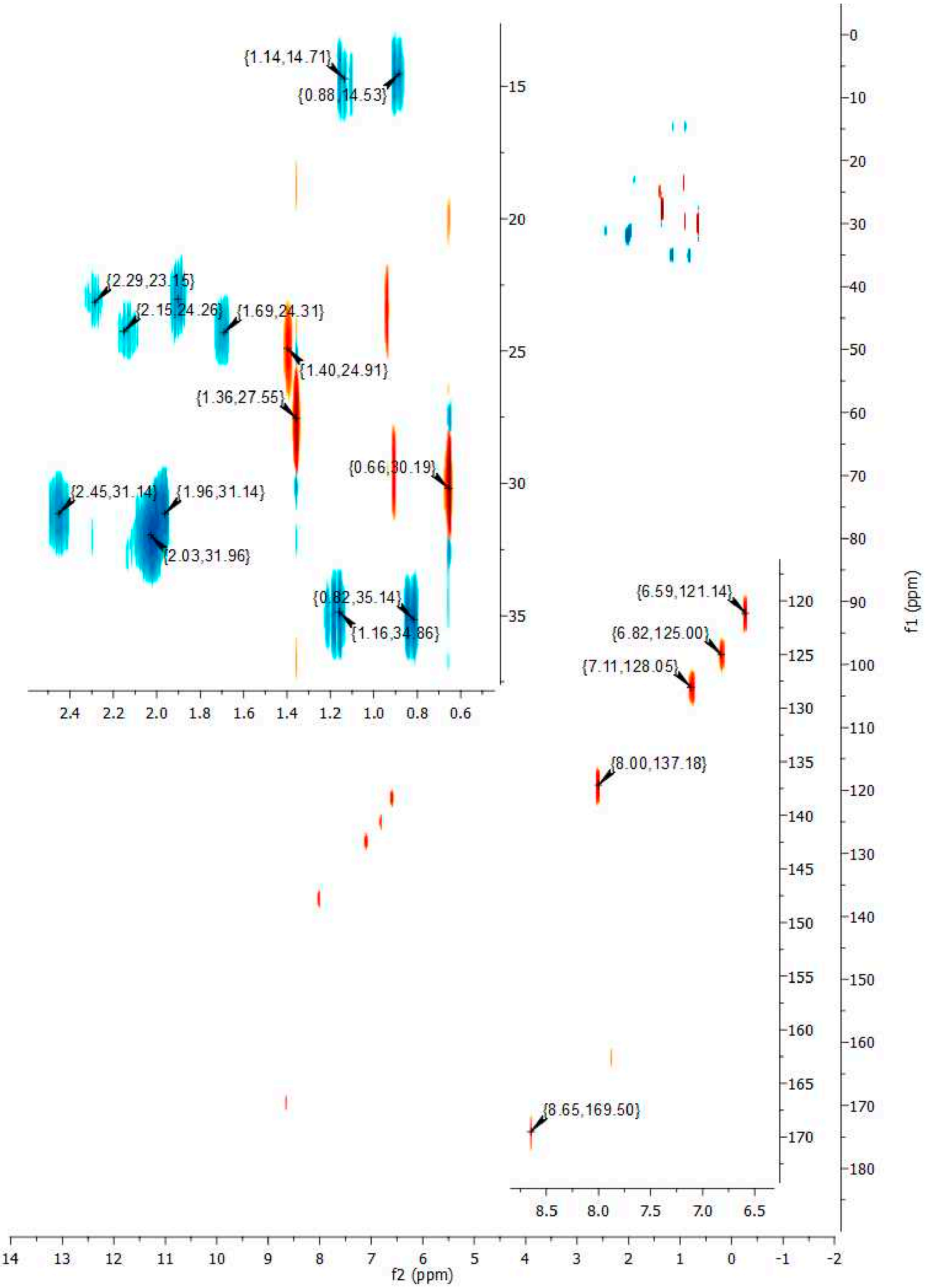

Figure S11. ^{13}C-^{1}H HSQC NMR (126 MHz–500 MHz, benzene-d_6) spectra of **3**.

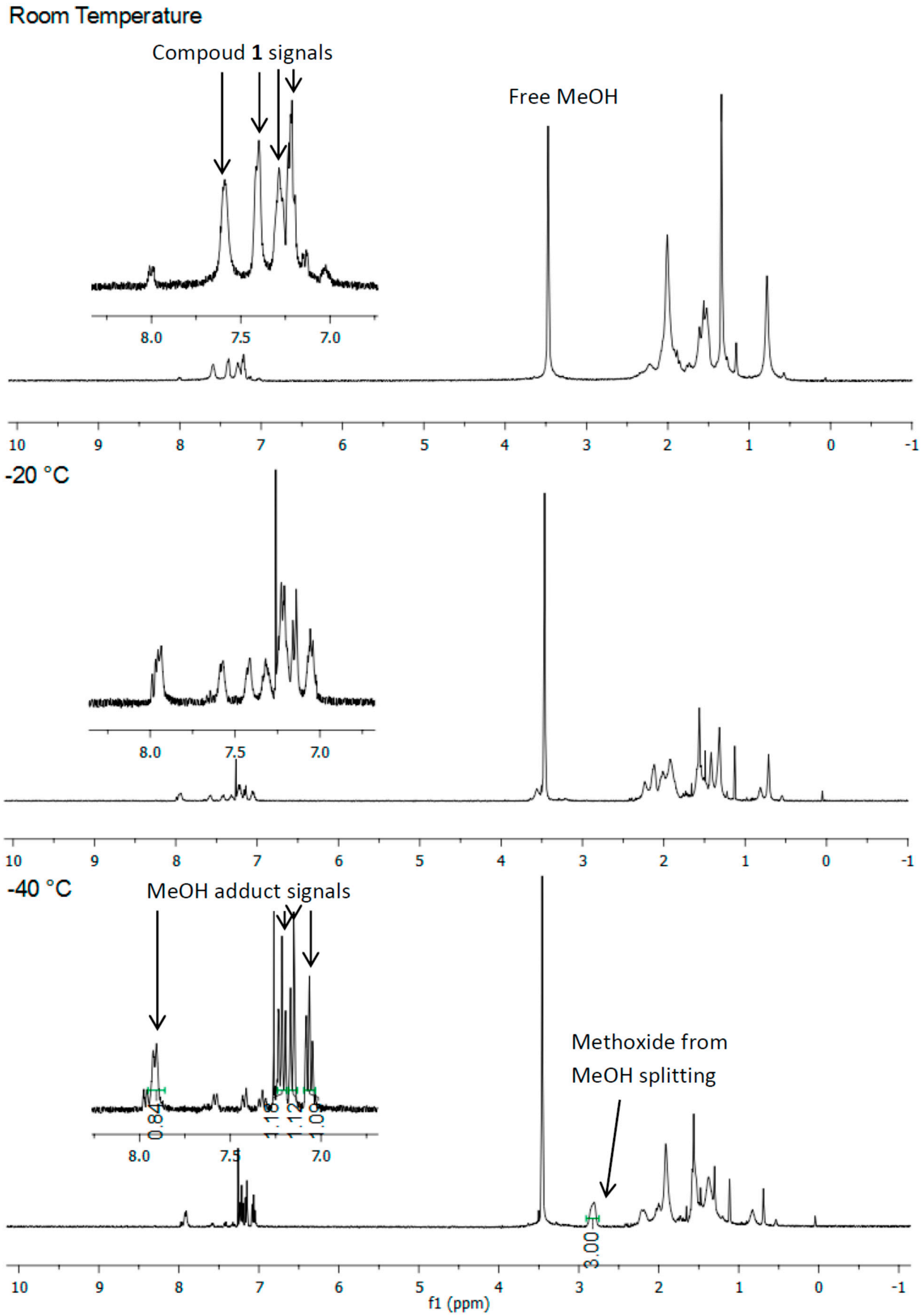

Figure S12. VT [1] H-NMR (400 MHz, $CDCl_3$) spectrum of **1** with *ca* 5eq of MeOH.

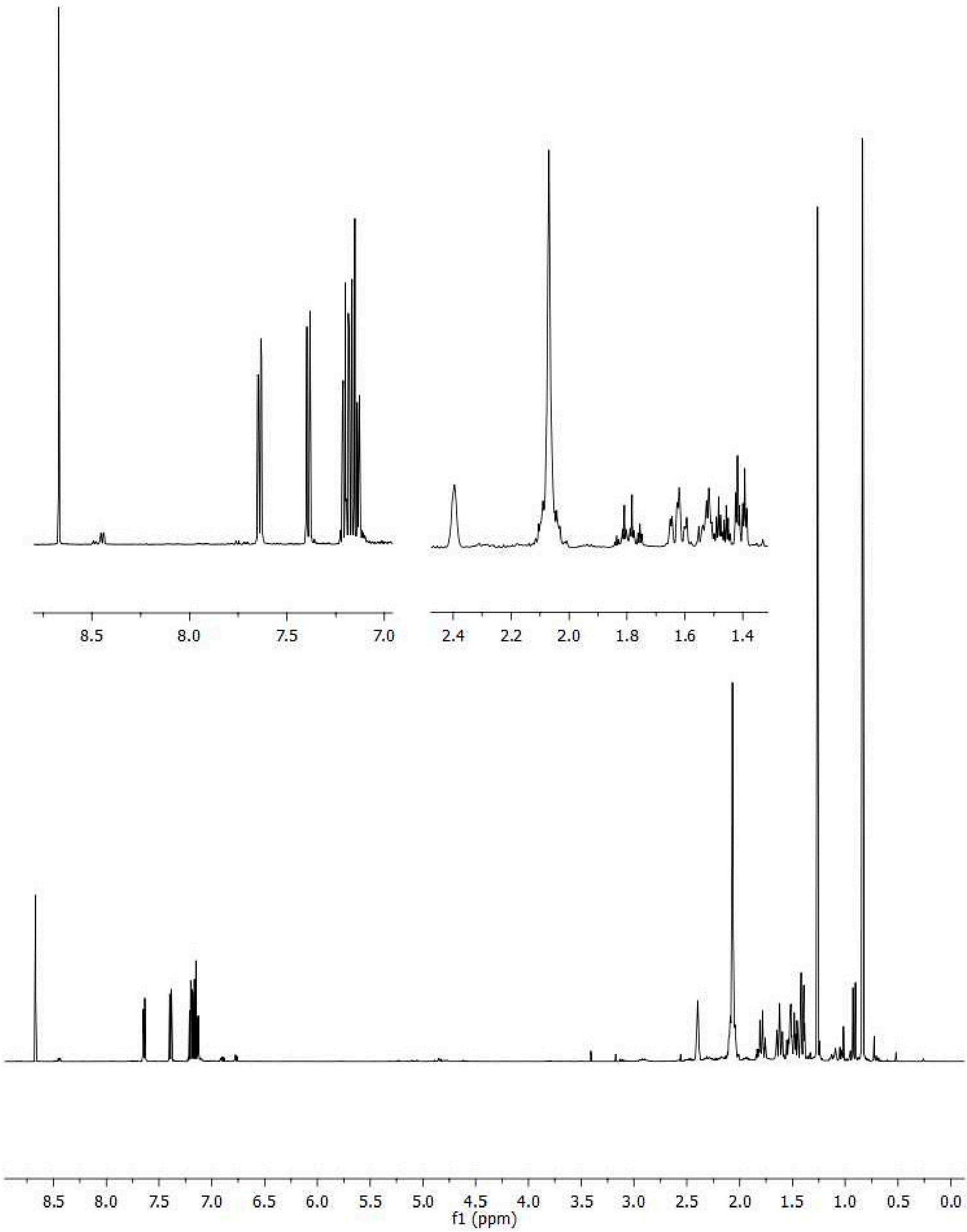

Figure S13. ^{1}H-NMR (500 MHz, benzene-d_6) spectra of **1** after heating with paraformaldehyde for 24 h.

S2 FTIR Spectra

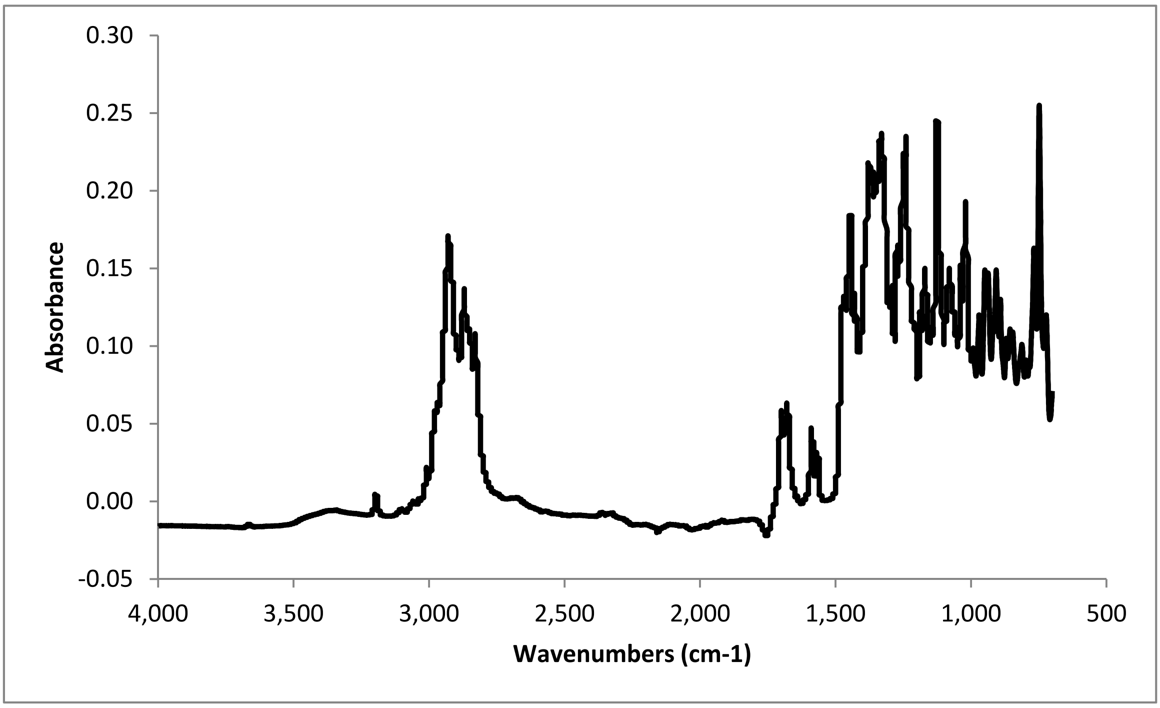

Figure S14. FTIR spectra of **1**.

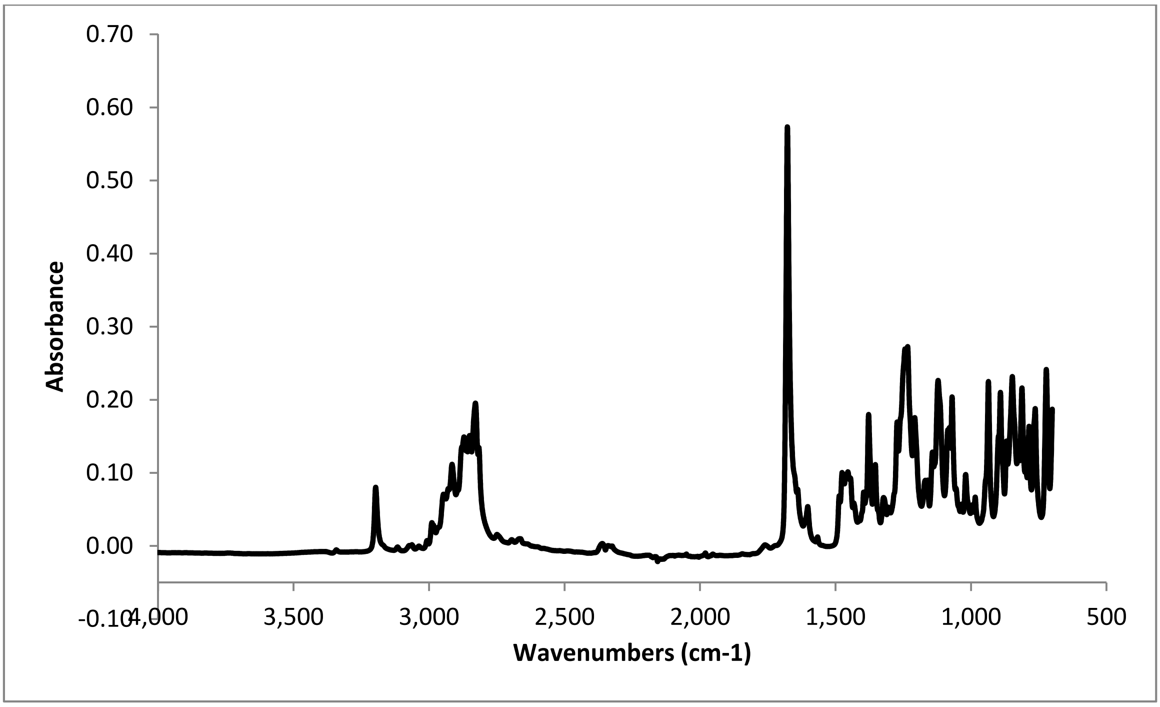

Figure S15. FTIR spectra of **3**.

S3 Calculations

General Information

All the calculations were performed on the full structures of the reported compounds. Calculations were performed with the GAUSSIAN 09 suite of programs [1]. The ωB97XD functional [2] was qualified as promising by Grimme [3] and was used to accurately describe the mechanism of FLP mediated hydrogenation of alkynes [4] and was thus used in combination with the 6-31G** basis set for all atoms [5,6]. The transition states were located and confirmed by frequency calculations (single imaginary frequency). The stationary points were characterized as minima by full vibration frequencies calculations (no imaginary frequency). All geometry optimizations were carried out without any symmetry constraints. The energies were then refined by single point calculations to include solvent effects using the SMD solvation model [7] with the experimental solvent (benzene) at the ωB97XD /6-31++G** level of theory [8]. All structures with their associated free enthalpy and Gibbs free energies as well as their cartesian coordinates are fully detailed in the following section.

1

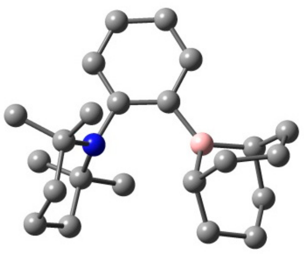

Sum of electronic and thermal Enthalpies= −976.987198
Sum of electronic and thermal Free Energies= −977.060951

C	−0.02940000	10.91230000	11.37160000
H	0.37370000	11.58720000	11.92390000
H	−0.83760000	10.60030000	11.78540000
H	−0.23390000	11.28360000	10.51020000
C	1.27590000	9.22900000	12.62580000
H	1.79470000	8.42330000	12.56420000
H	0.46080000	9.04770000	13.09900000
H	1.77810000	9.89800000	13.09630000
C	0.94400000	9.73920000	11.20420000
C	0.20580000	8.64670000	10.41170000
H	−0.46460000	8.24490000	10.98600000
H	−0.25630000	9.06100000	9.66550000
C	1.11240000	7.54980000	9.87620000
H	0.59710000	6.93340000	9.33370000
H	1.50110000	7.05480000	10.61440000
C	2.21650000	8.17600000	9.03660000
H	1.82010000	8.63400000	8.27930000
H	2.79170000	7.47430000	8.69200000
C	3.06420000	9.17370000	9.84260000
C	3.94310000	8.38290000	10.84550000
H	4.51540000	8.99080000	11.31780000
H	4.47880000	7.74570000	10.36760000
H	3.37900000	7.92220000	11.47140000
C	4.03190000	9.87090000	8.87600000
H	3.53170000	10.37440000	8.22990000
H	4.56330000	9.21090000	8.42460000
H	4.60550000	10.46170000	9.36940000
N	2.11940000	10.19430000	10.40260000
C	2.72210000	11.43650000	10.81900000
C	3.64780000	11.47370000	11.87290000
H	3.83420000	10.69140000	12.34070000
C	4.29290000	12.65110000	12.23500000
H	4.91970000	12.64820000	12.92210000
C	3.99760000	13.82550000	11.56860000
H	4.44410000	14.61260000	11.78270000
C	3.02670000	13.81580000	10.57560000
H	2.79990000	14.61830000	10.16600000
C	2.37130000	12.63890000	10.16360000
B	1.27550000	12.79380000	9.04190000
C	1.09250000	11.94080000	7.73980000
H	1.61760000	11.11650000	7.81720000
C	−0.38270000	11.58000000	7.47680000
H	−0.45070000	11.20570000	6.58450000
H	−0.64280000	10.88690000	8.10240000
C	−1.38180000	12.73190000	7.59370000
H	−1.35100000	13.25560000	6.77780000
H	−2.27560000	12.36350000	7.67320000
C	−1.12780000	13.65770000	8.77870000
H	−1.43910000	13.21760000	9.58460000
H	−1.66010000	14.46080000	8.66180000
C	0.34710000	14.07590000	8.98760000
H	0.42700000	14.56900000	9.83150000
C	0.89630000	14.95430000	7.82590000
H	0.28800000	15.69830000	7.68970000
H	1.75080000	15.32440000	8.09830000
C	1.08500000	14.23530000	6.49220000
H	1.65000000	14.77780000	5.91940000
H	0.22240000	14.14760000	6.05790000
C	1.71430000	12.84260000	6.63510000
H	2.66000000	12.95000000	6.82310000
H	1.63780000	12.38250000	5.78510000

H_2

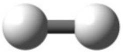

Sum of electronic and thermal Enthalpies=−1.161392

Sum of electronic and thermal Free Energies= −1.176183

H	2.78486400	0.63636400	0.00000000
H	2.04240800	0.63636400	0.00000000

CO_2

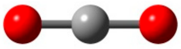

Sum of electronic and thermal Enthalpies= −188.512018

Sum of electronic and thermal Free Energies= −188.536311

C	0.00000000	0.00000000	0.00000000
O	1.16509200	0.00000000	0.00000000
O	−1.16509200	0.00000000	0.00000000

H_2O

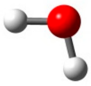

Sum of electronic and thermal Enthalpies= −76.388650

Sum of electronic and thermal Free Energies= −76.410723

O	1.69129100	−0.38241200	0.00000000
H	2.65004500	−0.33932300	0.00000000
H	1.41184600	0.53576200	0.00000000

MeOH

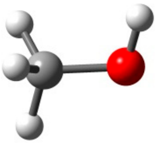

Sum of electronic and thermal Enthalpies= −115.642510

Sum of electronic and thermal Free Energies= −115.669602

C	1.04382800	−0.34318700	0.00001500
H	1.39202900	−1.37859800	−0.00002200
H	1.45662600	0.15126300	0.89143300
H	1.45656500	0.15130600	−0.89140700
O	−0.36445800	−0.38082300	0.00006200
H	−0.68595400	0.52317900	0.00009400

HCOOH

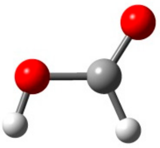

Sum of electronic and thermal Enthalpies= −189.673112

Sum of electronic and thermal Free Energies= −189.701396

C	−2.45213200	1.22360500	0.03489100
H	−3.45994100	0.78067000	−0.07696100
O	−1.95848300	1.49085500	1.08980600
O	−1.82326800	1.43662800	−1.13406800
H	−2.38460800	1.15653100	−1.86461500

CH_2O

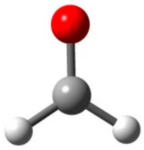

Sum of electronic and thermal Enthalpies= −114.441277
Sum of electronic and thermal Free Energies= −114.466744

C	−5.08872300	−1.59576700	0.02649700
H	−5.12834800	−2.70298200	−0.01622000
H	−5.12832600	−1.07917100	−0.95364700
O	−5.01005600	−0.99560100	1.06596900

1-H_2

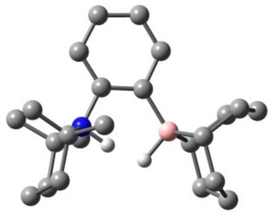

Sum of electronic and thermal Enthalpies= −978.152325
Sum of electronic and thermal Free Energies= −978.226136

C	1.62769700	5.99265400	1.22660300
C	1.17872100	5.17175500	2.27867300
C	2.67464100	6.86199600	1.59031100
C	1.71576300	5.20886800	3.55982300
H	0.37526900	4.47148700	2.08619000
C	3.23508400	6.92335700	2.86521900
C	2.75118900	6.08733200	3.86210400
H	1.32517500	4.54617200	4.32655700
H	4.03942100	7.61205300	3.09117100
H	3.18038900	6.12552600	4.85789100
C	2.76653800	9.22716700	0.63275300
C	4.55276700	7.38261000	−0.06330100
C	3.03024300	9.87527700	−0.73654100
C	4.69623700	8.14860900	−1.38932700
C	4.44139300	9.64613300	−1.26521700
H	2.81354500	10.94411300	−0.64418100
H	2.30957600	9.46715600	−1.45713800
H	5.69862300	7.95077000	−1.78171100
H	3.98492400	7.72594000	−2.11095100
H	4.54967400	10.12089300	−2.24478600
H	5.18455900	10.11577300	−0.61049800
N	3.16824500	7.74392700	0.51069100
C	3.48531600	9.98105700	1.75298000
H	3.07129700	10.99281000	1.78359100
H	3.29847100	9.52720700	2.72663000
H	4.56025900	10.07491900	1.60384200
C	5.71984400	7.67836300	0.88005400
H	5.62479700	7.13383700	1.81988100
H	6.63233400	7.32453000	0.39220000
H	5.85740000	8.73723100	1.09522400
C	1.25966400	9.26711100	0.91001800
H	0.69919600	8.69772000	0.16576300
H	1.01832600	8.86912300	1.89732300
H	0.93167400	10.30934000	0.86599200
C	4.54031000	5.87991600	−0.36497900
H	3.69784400	5.60463600	−1.00273600
H	5.46674700	5.62940600	−0.88917400
H	4.48787800	5.28367500	0.54786300
H	2.51582400	7.40042400	−0.26096900
H	1.60318200	6.77871100	−1.01001600
B	0.99469400	5.93399800	−0.28593900
C	1.15639400	4.50953400	−1.08081700
C	−0.58964600	6.32643900	−0.43116200
C	0.24666200	3.41834700	−0.48052800
C	0.87731500	4.77602100	−2.57440300
H	2.18119600	4.11150600	−1.01774100
C	−1.50005400	5.24114500	0.17744800
C	−0.86993300	6.58594100	−1.92626900
H	−0.83850900	7.25835300	0.10004900
H	0.65981400	3.13528800	0.49552200
C	−1.23750000	3.80240000	−0.31137400
H	0.29627900	2.50355700	−1.09022900
H	1.67292600	5.43634900	−2.94927200
H	0.94901400	3.84576800	−3.15816100
C	−0.48228900	5.43473100	−2.87385300
H	−2.55760700	5.47934000	−0.01140800
H	−1.38592900	5.27908900	1.26803500
H	−0.30430400	7.48253600	−2.21929900
H	−1.93157200	6.82577200	−2.08975400
H	−1.75342600	3.65169400	−1.26365000
H	−1.71098600	3.09694300	0.38402900
H	−1.26432800	4.67047500	−2.84899500
H	−0.47927500	5.80840600	−3.90611500

1-CO_2

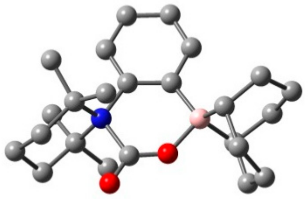

Sum of electronic and thermal Enthalpies= −1165.471444
Sum of electronic and thermal Free Energies= −1165.544557

C	−0.26334300	−0.65151600	1.31715400
C	−0.52945200	−1.24235800	2.57016200
C	0.76453600	0.30496900	1.29336900
C	0.15283000	−0.93575200	3.73150900
H	−1.31638800	−1.98158100	2.62506200
C	1.46007100	0.61901700	2.47201600

C	1.16662100	0.01196600	3.67945700
H	−0.10110900	−1.42866900	4.66473100
H	2.24319800	1.35237200	2.48060900
H	1.72902200	0.28717300	4.56562800
C	0.81308100	2.61522300	0.15253900
C	2.67106400	0.69178500	−0.40822300
C	1.46587400	3.42405800	−0.98979900
C	3.16429200	1.65044900	−1.51316000
C	2.94781700	3.12370700	−1.18548400
H	1.33164300	4.47912400	−0.73089300
H	0.93622000	3.25320900	−1.92326600
H	4.23305900	1.44908900	−1.63738800
H	2.68130800	1.41665100	−2.45909000
H	3.33199100	3.73481900	−2.00751300
H	3.52337300	3.41259900	−0.29749900
N	1.16387800	1.05281800	0.02481700
C	1.25359900	3.26806800	1.47315100
H	0.99296700	4.32552000	1.38598800
H	0.72569200	2.87694400	2.34032700
H	2.32945700	3.22381600	1.64800800
C	3.70336800	0.74562100	0.72952100
H	3.54062400	−0.01562000	1.48994300
H	4.67005300	0.53627600	0.26550100
H	3.78962300	1.72490700	1.20217400
C	−0.71603900	2.71855000	0.09698000
H	−1.12672600	2.43000800	−0.87175500
H	−1.18223000	2.11001800	0.87643200
H	−0.99261500	3.76162800	0.27039500
C	2.64295600	−0.75749000	−0.91196600
H	2.21086300	−1.43001200	−0.16586400
H	2.09664000	−0.86907600	−1.84961400
H	3.67342500	−1.07169900	−1.09564300
B	−1.13969600	−1.09992000	0.05021700
C	−1.13685900	−2.67431800	−0.37320700
C	−2.70281200	−0.63750100	0.04223100
C	−1.95800300	−3.52676300	0.61735000
C	−1.67599800	−2.78875800	−1.81768600
H	−0.11585100	−3.08695700	−0.37976000
C	−3.50068800	−1.45524600	1.07738700
C	−3.25533100	−0.77414600	−1.39370900
H	−2.81403000	0.42160400	0.32351500
H	−1.37999700	−3.64477100	1.54116000
C	−3.36744000	−2.98898400	0.95605100
H	−2.06921300	−4.54647900	0.22147300
H	−0.96063400	−2.30621400	−2.49292700
H	−1.71878200	−3.84573700	−2.11800300
C	−3.06403400	−2.15925000	−2.04065400
H	−4.56831000	−1.20053700	1.01323700
H	−3.18814600	−1.13855100	2.07993600
H	−2.75391600	−0.02817800	−2.02197300
H	−4.32571000	−0.52245200	−1.41233600
H	−4.07003000	−3.33984700	0.19605400
H	−3.70822900	−3.44960900	1.89216300
H	−3.83426400	−2.84309100	−1.67225000
H	−3.24579800	−2.07308500	−3.11904600
O	−0.46388900	−0.36251100	−1.14292300
C	0.32427700	0.59233900	−1.27709900
O	0.56882600	1.19363700	−2.29296600

1-H2O (2)

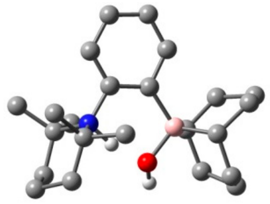

Sum of electronic and thermal Enthalpies= −1053.400185
Sum of electronic and thermal Free Energies= −1053.474466

C	0.24691600	3.08335500	0.20261800
C	0.88708800	4.17095800	−0.68241400
C	−0.91224400	5.87992100	0.09342700
C	−1.60113300	4.79658200	0.94708600
H	0.86536500	2.96949500	1.09951000
H	−2.69406200	4.89007100	0.86136600
H	−1.38321000	4.99883300	2.00157900
C	0.28229600	4.17769600	−2.10598600
H	0.91750800	4.78894900	−2.77078600
H	0.33294100	3.17133700	−2.54566900
C	−1.47634600	5.93159600	−1.34396100
H	−1.06175300	6.81839400	−1.83896200
H	1.94780700	3.89322300	−0.79393900
H	−1.15136300	6.84884900	0.55929600
C	−1.22135700	3.33453400	0.61332400
H	−1.87339600	2.97565400	−0.18771500
H	−1.46036600	2.69890000	1.47605200
H	0.29569200	2.10864500	−0.30498100
C	−1.16889800	4.68882400	−2.20114200
H	−1.84698900	3.88075600	−1.91417200
H	−1.40466700	4.90388000	−3.25136200
H	−2.56637200	6.07600000	−1.32417300
B	0.71159700	5.66652000	−0.02063400
C	1.56924300	5.90808900	1.38462200
C	1.26516200	5.15421400	2.53687400
C	2.62478200	6.82388800	1.57740400
C	1.92917900	5.28129400	3.74975900
H	0.46712500	4.42693700	2.48698900
C	3.30637400	6.97457600	2.78725900
C	2.96270400	6.20032100	3.88451900
H	1.63505600	4.66083900	4.59120800
H	4.10504800	7.69877300	2.87928100
H	3.49162800	6.31852100	4.82457800
O	1.24733500	6.72741500	−1.02432400
N	3.01900100	7.67690200	0.43365400
C	2.59328500	9.15188700	0.55023900
C	4.37201500	7.33149100	−0.20704500
H	2.32082100	7.31000900	−0.33447100
C	2.74994900	9.76708700	−0.85109900
C	3.36195500	9.96577100	1.59531700
C	1.10635900	9.17068100	0.93068300
C	4.42183300	8.06472300	−1.55991500
C	5.60357200	7.67101700	0.63773200
C	4.37424900	5.81931700	−0.46567100
C	4.13222700	9.55807600	−1.45914300
H	2.51154300	10.83279600	−0.77773200
H	1.99718700	9.31367700	−1.50720200
H	2.94583300	10.97728800	1.59252800
H	3.22247800	9.56403900	2.59928500
H	4.42902300	10.05432100	1.39357100
H	0.51355000	8.56423900	0.24323200
H	0.94600800	8.80333400	1.94634000
H	0.75333300	10.20474400	0.87939000
H	5.40518900	7.88369500	−2.00539000
H	3.68299500	7.60713300	−2.22962800
H	5.60990400	7.12243200	1.57978500
H	6.48618800	7.35431900	0.07432700
H	5.71712100	8.73422900	0.84571400
H	3.48697400	5.50945300	−1.02071200
H	5.26074200	5.56750400	−1.05462400
H	4.40512200	5.25225800	0.46672500
H	4.17010800	10.00913600	−2.45530900
H	4.90118100	10.06341600	−0.86324800
H	1.29654400	6.36264500	−1.90879700

1-MeOH

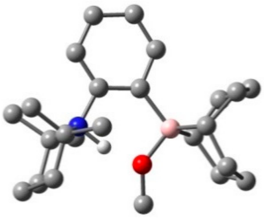

Sum of electronic and thermal Enthalpies= −1092.646982
Sum of electronic and thermal Free Energies= −1092.724062

C	0.29885000	3.08122600	0.18710700
C	0.80362700	4.14277700	−0.81099500
C	−0.89819400	5.88153500	0.10252000
C	−1.53553600	4.79716900	1.00130900
H	1.00141000	3.00599800	1.02194700
H	−2.63169800	4.86751000	0.93765200
H	−1.30179500	5.03173600	2.04438200
C	−0.02347600	4.02978500	−2.12153200
H	0.54854900	4.44762200	−2.95249500
H	−0.13937200	2.96558000	−2.37420700
C	−1.59430500	5.94695100	−1.26856300
H	−1.20814300	6.81543400	−1.81432200
H	1.83974100	3.86623900	−1.06592800
H	−1.07959600	6.84812500	0.59774900
C	−1.13860100	3.32804800	0.69966100
H	−1.83311500	2.92308900	−0.04196300
H	−1.30613200	2.72408200	1.60120000
H	0.31749100	2.09092600	−0.29070100
C	−1.43450600	4.66737700	−2.11068600
H	−2.15642700	3.93066300	−1.74564900
H	−1.73787400	4.87302800	−3.14563200
H	−2.67045500	6.13550600	−1.14008200
B	0.70745700	5.64275400	−0.13755800
C	1.59410800	5.87377800	1.27494800
C	1.30259500	5.10061500	2.42079900
C	2.60652800	6.82905300	1.50994900
C	1.93295000	5.23780500	3.64972700
H	0.53511200	4.34600400	2.36108600
C	3.25577300	6.99095500	2.73732000
C	2.92507600	6.19396800	3.82013800
H	1.64127800	4.59472200	4.47482100
H	4.02399000	7.74304900	2.85208300
H	3.43020400	6.32312600	4.77170600
O	1.23877200	6.77757000	−1.09406200
N	3.00794300	7.71412500	0.39413500
C	2.58180800	9.18556300	0.54753200
C	4.38271500	7.38599800	−0.21051600
H	2.29859400	7.35926400	−0.38161100
C	2.79974600	9.85339400	−0.82057300
C	3.30727700	9.96795800	1.64784000
C	1.08150300	9.19401000	0.87116800
C	4.51408000	8.18131800	−1.52153100
C	5.58271300	7.67241900	0.69915300
C	4.38332200	5.88539200	−0.53276400
C	4.20870900	9.66786400	−1.37347200
H	2.55980900	10.91611600	−0.71629600
H	2.07379100	9.43704000	−1.52876800
H	2.92245600	10.99166200	1.62649400
H	3.09228200	9.56547000	2.63761400
H	4.38723400	10.02520700	1.51518600
H	0.51523600	8.62000700	0.13526300
H	0.88429800	8.77605900	1.86055100
H	0.73193000	10.23040500	0.85919100
H	5.52841400	8.02868800	−1.90364700
H	3.83617100	7.75029700	−2.26506900
H	5.54905900	7.08285700	1.61505300
H	6.48298200	7.37141200	0.15565300
H	5.69907500	8.72459200	0.95769900
H	3.50216700	5.59429700	−1.10737000
H	5.27563100	5.65590100	−1.12206000
H	4.40447900	5.28081300	0.37607300
H	4.29184800	10.15846900	−2.34800500
H	4.94526700	10.15172000	−0.72165400
C	1.38791100	6.73130100	−2.48477700
H	2.09407600	5.95370200	−2.81077600
H	0.43697700	6.56550100	−2.99738600
H	1.77315400	7.70062900	−2.82813000

1-HCOOH (3)

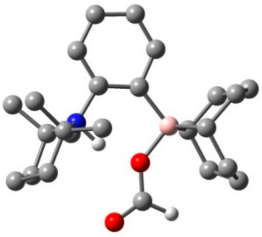

Sum of electronic and thermal Enthalpies= −1166.690769
Sum of electronic and thermal Free Energies= −1166.768467

C	0.22839500	3.03636600	0.29520800
C	0.82789800	4.07275700	−0.68066800
C	−0.95285100	5.82708400	0.05286800
C	−1.57514600	4.80833700	1.03260000
H	0.89193100	2.93924800	1.15952900
H	−2.67028100	4.90040000	1.00309000
H	−1.29973600	5.08314800	2.05500300
C	0.16234600	3.94951400	−2.06775000
H	0.78983400	4.45942900	−2.80648800
H	0.16485500	2.89697300	−2.38456300
C	−1.63991900	5.72692000	−1.32670500
H	−1.41370800	6.63217400	−1.90006400
H	1.88778600	3.80664900	−0.81283800
H	−1.17990200	6.82814300	0.45018400
C	−1.21731500	3.32767400	0.76205200
H	−1.90831700	2.93903400	0.00884700
H	−1.42517900	2.74100600	1.66614100
H	0.23537100	2.04478000	−0.17908500

C	−1.29309700	4.46786000	−2.15691700
H	−1.96406500	3.66099600	−1.85045700
H	−1.54066200	4.65507800	−3.21007000
H	−2.73082600	5.75254800	−1.19449000
B	0.66013100	5.59498300	−0.10360800
C	1.56195500	5.91843500	1.26909300
C	1.27750400	5.16214200	2.42834200
C	2.60602700	6.84828500	1.47319400
C	1.93190500	5.29208900	3.64451000
H	0.49700700	4.42067300	2.38615100
C	3.28211800	6.99769900	2.68766300
C	2.95206300	6.22197000	3.78519100
H	1.63978300	4.66239200	4.47942900
H	4.07353500	7.72700300	2.78093800
H	3.48092200	6.34675700	4.72416100
O	1.24814400	6.64229800	−1.17277300
C	1.05130600	6.96507500	−2.42813500
H	0.27551400	6.39975600	−2.95471600
O	1.68460500	7.83934900	−2.99057600
N	3.03397400	7.73253600	0.36031300
C	2.63140800	9.22089300	0.53433800
C	4.41916800	7.38031800	−0.24271400
H	2.38453200	7.42712900	−0.40933100
C	2.94040700	9.93469000	−0.79174200
C	3.31199700	9.93773200	1.70369400
C	1.11651300	9.23960200	0.77364100
C	4.59316400	8.23225700	−1.51049300
C	5.60087800	7.57598200	0.71103500
C	4.36267500	5.89941100	−0.63780500
C	4.36843400	9.72241300	−1.28134200
H	2.72714000	10.99844600	−0.64648100
H	2.25704400	9.56300100	−1.56187500
H	2.98759800	10.98156200	1.67216800
H	3.00417400	9.53286300	2.66769700
H	4.40041100	9.93688300	1.64677300
H	0.57825900	8.71486800	−0.01837200
H	0.85190400	8.78355700	1.72992200
H	0.78433700	10.28124500	0.78420900
H	5.59794600	8.03916900	−1.89946100
H	3.87687100	7.89307000	−2.26552800
H	5.57167600	6.87479200	1.54526100
H	6.51117000	7.36443500	0.14317900
H	5.69318400	8.58951900	1.10117100
H	3.52323600	5.69168000	−1.30431200
H	5.28874000	5.65032200	−1.16306100
H	4.27691700	5.24969700	0.23579700
H	4.51125300	10.25778200	−2.22425000
H	5.09971100	10.13298700	−0.5740060

1-CH_2O

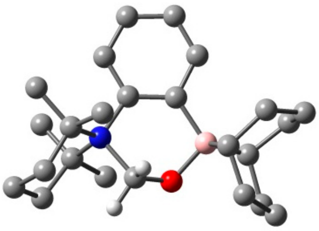

Sum of electronic and thermal Enthalpies= −1091.430292
Sum of electronic and thermal Free Energies= −1091.503354

C	−0.24072300	−0.71235900	1.33504800
C	−0.54624800	−1.28199500	2.58684100
C	0.78211700	0.25865000	1.35490400
C	0.11228400	−0.97266400	3.76484500
H	−1.34045200	−2.01528800	2.62798400
C	1.47488800	0.55897700	2.53586500
C	1.15049000	−0.05060800	3.73657800
H	−0.17310700	−1.45602200	4.69436200
H	2.27602800	1.27657600	2.54712400
H	1.70342500	0.20404600	4.63500300
C	0.82022600	2.59846400	0.31618200
C	2.59977900	0.73487700	−0.41586700
C	1.17413100	3.32983600	−0.99223000
C	2.92275800	1.64843500	−1.62094900
C	2.62826600	3.12919400	−1.40534500
H	0.96410700	4.39151000	−0.82972600
H	0.50782300	3.01797800	−1.80162000
H	3.98439900	1.50497700	−1.84242400
H	2.39493300	1.29951600	−2.51291300
H	2.83049400	3.67744900	−2.33034700
H	3.29980400	3.54741500	−0.64723100
N	1.13644000	1.05280900	0.11529200
C	1.56876700	3.29806800	1.46920400
H	1.39106200	4.36952000	1.34270000
H	1.16562600	3.01764100	2.44081600
H	2.64586200	3.15932600	1.47670700
C	3.72613700	0.88499600	0.62394500
H	3.64425700	0.14697000	1.42005900
H	4.66059200	0.68015700	0.09585300
H	3.82827000	1.87654700	1.05919100
C	−0.67436100	2.79276900	0.65042400
H	−1.32607000	2.79640100	−0.22397400
H	−0.76817700	1.17024300	−0.88976300
H	−1.03077600	2.03240900	1.34903600
H	−0.78866600	3.76669000	1.13230100
C	2.66446800	−0.73459800	−0.86378800
H	2.35363900	−1.40554500	−0.06036300
H	2.04400000	−0.95360400	−1.72895000
H	3.70757400	−0.95155600	−1.11144700
B	−1.00677600	−1.21030100	−0.01883300
C	−1.15423000	−2.81885300	−0.27111800
C	−2.52333400	−0.60925500	−0.19934500
C	−2.16294600	−3.50048900	0.67674900
C	−1.54992200	−3.00628800	−1.75340600
H	−0.18790400	−3.32811300	−0.13300200
C	−3.46040400	−1.23687100	0.85119800
C	−2.97300200	−0.84062900	−1.65708600
H	−2.57724600	0.47918900	−0.02427700
H	−1.69164100	−3.62956000	1.65776700
C	−3.51767400	−2.77758600	0.85244500
H	−2.36536200	−4.52463200	0.32921600
H	−0.73215300	−2.62860200	−2.37645400
H	−1.65395200	−4.07669100	−1.98558100
C	−2.85120400	−2.29169100	−2.16134400
H	−4.48498800	−0.85675800	0.72447500
H	−3.13827300	−0.88662100	1.84090000
H	−2.35310900	−0.20463300	−2.30401000
H	−4.01003500	−0.50206800	−1.80200800
H	−4.20105300	−3.10085500	0.06242100
H	−3.98433800	−3.11455100	1.78726400
H	−3.70823800	−2.87160500	−1.80638100
H	−2.93544000	−2.29525000	−3.25584300
O	−0.08174200	−0.70907900	−1.12843900
C	0.13169100	0.59093900	−1.08283400
H	0.62409300	0.94837800	−1.98441700

S4 Crystallographic data

General Information

Single crystals with suitable size of all compounds were mounted on CryoLoops with Paratone-N and optically aligned on a Bruker SMART APEX−II X−ray diffractometer with 1K CCD detector using a digital camera. Initial intensity measurements were performed using a fine−focused sealed tube, graphite-monochromated, X-ray source (Mo Kα, λ = 0.71073 Å) at 50 kV and 30 mA. Standard APEX-II [9] software package was used for determining the unit cells, generating the data collection strategy, and controlling data collection. SAINT [10] was used for data integration including Lorentz and polarization corrections. Semi-empirical absorption corrections were applied using SCALE (SADABS) [11]. The structures of all compounds were solved by direct methods and refined by full-matrix least-squares methods with SHELX-97 [12] in the SHELXTL6.14 package. As the solvent molecules in some compounds are highly disordered, the SQUEEZE subroutine of the PLATON [13] software suit was used to remove the scattering contributions from the highly disordered guest molecules. The resulting new HKL files were used to further refine the structures. All of the H atoms (on C atoms) were generated geometrically and refined in riding mode. Crystallographic information for all obtained phases is summarized in Table S1. Atomic coordinates and additional structural information are provided in the cif files of the Supporting Information.

Table S1. Crystal data and structure refinement for **1** and **2**.

	**1**	**2**
Empirical formula	C23 H36 B N	C23 H38 B N O
Formula weight	337.34	355.35
Temperature	150(2) K	296(2) K
Wavelength	0.71073 Å	0.71073 Å
Crystal system	Monoclinic	Orthorhombic
Space group	$P2_1/c$	$P2_12_12_1$
Unit cell dimensions	a = 7.6005(6) Å	a = 11.546(4) Å
	b = 18.9799(16) Å	b = 13.277(5) Å
	c = 13.7261(12) Å	c = 13.602(5) Å
	α = 90°	α = 90°
	β = 92.6030(10)°	β = 90°
	γ = 90°	γ = 90°
Volume	1978.0(3) $Å^3$	2085.1(14) $Å^3$
Z	4	4
Density (calculated)	1.133 Mg/m^3	1.132 Mg/m^3
Absorption coefficient	0.063 mm^{-1}	0.067 mm^{-1}
F(000)	744	784
Crystal size	0.580 × 0.460 × 0.280 mm^3	0.480 × 0.300 × 0.220 mm^3
Theta range for data collection	1.832 to 28.215°	2.144 to 30.530°
Index ranges	$-10 \le h \le 10, -25 \le k \le 25, -18 \le l \le 18$	$-14 \le h \le 16, -16 \le k \le 18, -19 \le l \le 19$
Reflections collected	20848	16231
Independent reflections	4871 [R(int) = 0.0300]	6365 [R(int) = 0.0200]
Completeness (theta = 25.242°)	100.0 %	100.0 %
Max. and min. transmission	0.982 and 0.965	0.985 and 0.976
Data/restraints/parameters	4871/0/230	6365/0/244
Goodness-of-fit on F^2	1.043	1.051
Final R indices [I > 2sigma(I)]	R1 = 0.0434, wR2 = 0.1160	R1 = 0.0354, wR2 = 0.0891
R indices (all data)	R1 = 0.0470, wR2 = 0.1193	R1 = 0.0394, wR2 = 0.0919
Largest diff. peak and hole	0.420 and −0.195 $e \cdot Å^{-3}$	0.307 and −0.163 $e \cdot Å^{-3}$

References

1. Frisch, M.J.; Trucks, G.W.; Schlegel, H.B.; Scuseria, G.E.; Robb, M.A.; Cheeseman, J.R.; Scalmani, G.; Barone, V.; Mennucci, B.; Petersson, G.A.; *et al. Gaussian 09*, revision C.01; Gaussian, Inc.: Wallingford, CT, USA, 2009.
2. Chai, J.D.; Head-Gordon, M. Long-range corrected hybrid density functionals with damped atom-atom dispersion corrections. *Phys. Chem. Chem. Phys.* **2008**, *10*, 6615–6620.
3. Goerigk, L.; Grimme, S. A thorough benchmark of density functional methods for general main group thermochemistry, kinetics, and noncovalent interactions. *Phys. Chem. Chem. Phys.* **2011**, *13*, 6670–6688.
4. Chernichenko, K.; Madarász, Á.; Pápai, I.; Nieger, M.; Leskelä, M.; Repo, T. A frustrated-Lewis-pair approach to catalytic reduction of alkynes to *cis*-alkenes. *Nat. Chem.* **2013**, *5*, 718–723.
5. Francl, M.M.; Pietro, W.J.; Hehre, W.J.; Binkley, J.S.; Gordon, M.S.; Defrees, D.J.; Pople, J.A. Self-consistent molecular orbital methods. XXIII. A polarization-type basis set for second-row elements. *J. Chem. Phys.* **1982**, *77*, 3654–3665.
6. Hehre, W.J.; Ditchfield, R.; Pople, J.A. Self-Consistent Molecular Orbital Methods. XII. Further Extensions of Gaussian-Type Basis Sets for Use in Molecular Orbital Studies of Organic Molecules. *J. Chem. Phys.* **1972**, *56*, 2257–2261.
7. Marenich, A.V.; Cramer, C.J.; Truhlar, D.G. Universal solvation model based on solute electron density and on a continuum model of the solvent defined by the bulk dielectric constant and atomic surface tensions. *J. Phys. Chem. B* **2009**, *113*, 6378–6396.
8. Grimme, S. Semiempirical GGA-type density functional constructed with a long-range dispersion correction. *J. Comp. Chem.* **2006**, *27*, 1787–1799.
9. *APEX2*; Bruker AXS Inc.: Madison, WI, USA, 2007.
10. *SAINT*; Bruker AXS Inc.: Madison, WI, USA, 2007.
11. Sheldrick, G.M. *SADABS*; Bruker AXS Inc.: Madison, WI, USA, 2007.
12. Sheldrick, G.M. A short history of SHELX. *Acta Crystallogr.* **2008**, *A64*, 112–122.
13. Spek, A.L. Structure validation in chemical crystallography. *Acta Crystallogr.* **2009**, *D65*, 148–155.
